# Distinct signatures of gut microbiota and metabolites in primary biliary cholangitis with poor biochemical response after ursodeoxycholic acid treatment

**DOI:** 10.1186/s13578-024-01253-1

**Published:** 2024-06-15

**Authors:** Weijia Han, Ting Song, Zhongyi Huang, Yanmin Liu, Bin Xu, Chunyang Huang

**Affiliations:** 1 Department of Gastroenterology, Shenzhen Hospital, Southern Medical University, Shenzhen, Guangdong China; 2 Second Department of Liver Disease Center, Beijing Youan Hospital, Capital Medical University, Beijing, China; 3 Department of Hepatology, The Sixth People’s Hospital of Qingdao, Qingdao, 266033 Shandong China; 4 Emergency Department, Shenzhen Hospital, Southern Medical University, Shenzhen, Guangdong China

## Abstract

**Background:**

About 1/3 of primary biliary cholangitis (PBC) patients suffered from poor response worldwide. And these patients present intestinal disturbances. We aimed to identify signatures of microbiota and metabolites in PBC patients with poor response, comparing to patients with response.

**Methods:**

This study enrolled 25 subjects (14 PBC patients with response and 11 PBC patients with poor response). Metatranscriptomics and metabolomics analysis were carried out on their fecal.

**Results:**

PBC patients with poor response had significant differences in the composition of bacteria, characterized by decreased *Gemmiger *etc*.* and increased *Ruminococcus *etc. The differential microbiota functions characterized by decreased abundance of elongation factor Tu and elongation factor G base on the KO database, as well as decreased abundance of Replicase large subunit etc*.* based on the SWISS-PROT database. PBC with poor response also had significant differences in 17 kinds of bacterial metabolites, characterized by decreased level of metabolites vital in bile acids metabolism pathway (L-Cysteine etc.) and the all-trans-Retinoic acid, a kind of immune related metabolite. The altered microbiota was associated with the differential expressed metabolites and clinical liver function indicators. 1 bacterial genera, 2 bacterial species and 9 metabolites simultaneously discriminated PBC with poor response from PBC with response with high accuracy.

**Conclusion:**

PBC patients with poor response exhibit unique changes in microbiota and metabolite. Gut microbiota and metabolite-based algorithms could be used as additional tools for differential prediction of PBC with poor prognosis.

**Supplementary Information:**

The online version contains supplementary material available at 10.1186/s13578-024-01253-1.

## Introduction

Primary biliary cholangitis (PBC) is a chronic cholestatic immune liver disease characterized by persistent cholestasis, interlobular bile duct damage, liver fibrosis, eventual cirrhosis, and death [1]. Its prevalence in Europe has an overall rate of 22.27 cases per 100,000 inhabitants and a pooled incidence rate of 1.7 new cases per 100,000 inhabitants per year, with a female predominance (1.6–4.8:1 female: male ratio) [2]. There are still approximately 30% of patients with PBC who are not responsive to ursodesoxycholic acid (UDCA), which is the only drug currently approved by the FDA for application in cholestatic liver disease [3]. But there is no evidence that glucocorticoids and immunosuppressive agents can be used directly used to treat PBC [4]. It is necessary to explore the other effective treatment for PBC with poor response.

Recent studies have suggested the adverse effect of microbiota dysbiosis in the treatment of PBC. Patients with PBC were depleted of some potentially beneficial bacteria, such as *Bacteroides* et al., but were enriched in some bacterial taxa containing opportunistic pathogens, such as *γ-Proteobacteria* et al. [5] Tang et al. suggested a reduced microbial diversity, as well as increased abundance of eight genera and decreased abundance of four genera in PBC [6]. Kitahata et al. also suggested dysbiosis of small intestinal mucosa-associated microbiota in PBC [7]. In previous study, we found lower bacterial diversity and lower abundance of 4 genera, including *Gemmiger*, *Blautia*, *Anaerostipes* and *Coprococcus* in PBC patients with poor biochemical response after UDCA treatment [8]. Luo et al. suggested the potential effect of microbial targets for the prevention and treatment of PBC [9]. However, the altered gut microbiota and microbiota function in PBC with poor response has not been elucidated, systemically.

The main characteristics of PBC are cholestasis caused by abnormal bile acids (BAs) metabolism and interlobular bile duct damage caused by abnormal immune response [1]. Studies had suggested the critical role of microbiome by interacting with BAs metabolism and immunity. The gut microbiota could interact with the host tissue cells, such as immune cells directly. The gut bacterial metabolites can also mediate host immunological and physipathological responses in the intestine and at distant organs [5]. In addition, the altered microbe-derived metabolites, such as valeric acid, are related to the enterohepatic circulation of BAs [10]. In previous study, we also found the relationship between altered microbiota genera and total BAs level in PBC patients with poor response [8]. So, integrated analysis of multiomics data from the gut microbiome and metabolome need to be conducted to identify the physiological effected by microbiota in PBC patients with poor biomedical response.

To bridge the abovementioned knowledge gaps, we applied metatranscriptomics analysis to elucidate the altered species and overall physiological process mediated by the microbiota in PBC patients with poor response. Then, we applied metabolomics analysis to elucidate the metabolites mediated by the gut microbiota in PBC patients with poor response. Furthermore, we clarified the relationship among clinic indicators, gut microbiota and metabolites. Finally, we identified classifiers that discriminates PBC patients with poor response from PBC patients with response. By utilizing multi-omics analyses, we characterized the biological landscape in the gut microbiome, microbial function and BAs metabolism and their interactions in PBC. We showed the microbial metabolic perturbations in PBC. We hope to provide a foundation for future therapies for the treatment of PBC with poor response.

## Methods and patients

### Study cohort

The study was approved by the ethics committee of Beijing Youan Hospital (approval no. LL-2018-044-K). This prospective observational study was carried out between October 2021 and October 2022 at Beijing YouAn Hospital, Beijing, China.

Similar to our previous study, based on the total bilirubin (TBIL), PBC patients were enrolled and divided into the response (TBIL ≤ 1 × ULN) and the poor response (TBIL > 1 × ULN) groups [8]. Stool sample analysis was performed after 12 months of UDCA treatment. UDCA at a dose of 13–15 mg/kg/day is the first-line therapy for PBC. (Supplementary Fig. 1).

The inclusion criteria were as follows: (1) patients diagnosed with PBC according to the 2017 European Association for the Diagnosis and Treatment of PBC [11]; (2) age > 18 years; (3) Chinese; (4) treated with UDCA for 12 months; (5) no alcohol consumption; and (6) no other chronic diseases. The exclusion criteria were as follows: (1) liver diseases caused by hepatitis virus, drugs, or alcoholic or nonalcoholic fatty liver disease; (2) hepatocellular carcinoma or liver metastases; (3) severe cardiac and renal insufficiency; (4) intestinal diseases or history of intestinal surgery; (5) use of antibiotics or intestinal microecologics within 2 weeks prior to enrollment; (6) the lack of compliance; (7) use of proton pump inhibitors; (8) autoimmune hepatitis; and (9) smoking.

Stool samples were collected and stored at − 80 °C. Written, informed consent was obtained from each study participant. At the same time, blood samples were collected and the serum was obtained for analysis of laboratory parameters including alanine transaminase (ALT), aspartate transaminase (AST), TBIL, albumin (ALB); alkaline phosphatase (ALP), gamma-glutamyl transpeptidase (GGT) and total BAs.

### Metatranscriptomics sequencing in stools

To obtain clean data, the original sequencing data were processed as follows: (1) Excluding Reads containing 10% uncertain bases; (2) Excluding Reads containing adapter sequences; (3) Excluding Reads containing low-quality bases of 20%; (4) For the sample with the host or environment sources, a filtering step is added here to remove the sequence of the host genome so as to reduce the interference of the host sequence on the subsequent analysis. High-quality short reads of each DNA sample were assembled by the IDBA [12]. Functional annotations were made by Diamond (v0.8.23.85) BLASTP search against KEGG and SwissProt databases. KEGG is a database resource for understanding high-level functions and utilities of the biological system. KEGG Orthology (KO) represented molecular functions in terms of functional orthologs. SWISS-PROT is a protein database describes the function of proteins, post-translational processing modifications of proteins, domains and binding sites of the proteins, and sequence similarity of proteins. Taxonomy annotation and abundance calculation was carried out through Kraken2. Kraken2 is the newest version of Kraken, a taxonomic classification system using exact k-mer matches to achieve high accuracy and fast classification speeds. Top 20 genes from annotation result (sorted by gene abundance in descending order) are depicted on circos plot, where distribution of functions in groups. (Supplementary method).

### Metabolomics analysis using stools

Stool samples were used for metabolomics analysis. A sample (100 mg) was weighed precisely and mixed with 800 μL of extract (methanol:acetonitrile:water = 2:2:1 (v:v:v), pre-cooled at − 20 °C). Then, the sample was homogenized at 50 Hz for 5 min by a tissue lyser with two glass beads. Then, 10 min of ultrasound agitation in water at 4 °C, and for 1 h at − 20 °C, was undertaken. After centrifugation at 25,000 rpm for 15 min at room temperature, the sample was mixed with 600 μL of complexation solution (methanol:H2O = 1:9 (v:v), followed by vortex-mixing for 1 min, ultrasound agitation in water for 10 min at 4 °C, and centrifugation at 25,000 rpm for 15 min at 4 °C. Then, two-dimensional ultra-high pressure liquid chromatography was done using a Waters system (Waltham, MA, USA). Tandem mass spectrometry was carried out using an Q Exactive™ high-resolution mass spectrometer (Thermo Fisher Scientific, Waltham, MA, USA) for the separation and detection of metabolites. The abundance of BAs ions analyzed in pos or neg mode was selected for comparison by rank sum test. (Supplementary method).

### Statistical analysis

Shannon index is calculated as: H = − ∑ iAi*ln (Ai) (Ai is the relative abundance of species i). Simpson diversity is calculated as: ${\text{D}} = 1 - \sum\nolimits_{i}^{S} {\frac{{n^{2} }}{{N^{2} }}}$ (D is the diversity index. N is the total number of individuals in all species. n is the total number of individuals of species i. S means sorts of species). Chao1 index is calculated as: ${\text{chao}}1 = {\text{S}} - \frac{{F_{1}^{2} }}{{2*F_{2} }}$ (S is the number of species observed in the sample. F1 and F2 represent the number of singletons and double sets, respectively). β diversity was evaluated using QIIME1 and the R v3.2.1 ggplot package; α diversity was evaluated using mothur-1.39.5/mothur and R ggplot package. principal coordinates analysis (PCoA) was performed using the mixOmics package of R. Identified species and predicted functional pathways were screened with the Wilcoxon test (P < 0.05 and |log [fold change]|> 1) using PICRUSt2 v2.2.0-b and R v.3.4.10. Metabolic pathway enrichment analysis of differential metabolites was based on the KEGG database. The metabolic pathways with p value < 0.05 were defined as the metabolic pathways with significant enrichment of differential metabolites. Spearman correlation coefficient analysis was performed to examine patterns, with P values < 0.05 considered statistically significant. Continuous variables were expressed as means and standard deviations. The Cytoscape_v3.2.1 was performed to show the metabolites and pathways. Clinic statistical analyses and Spearman analysis were performed using the SPSS software (ver. 19), with P values < 0.05 considered statistically significant. (Supplementary method).

## Results

### Characteristics of the entire cohort

**Fig. 1:**
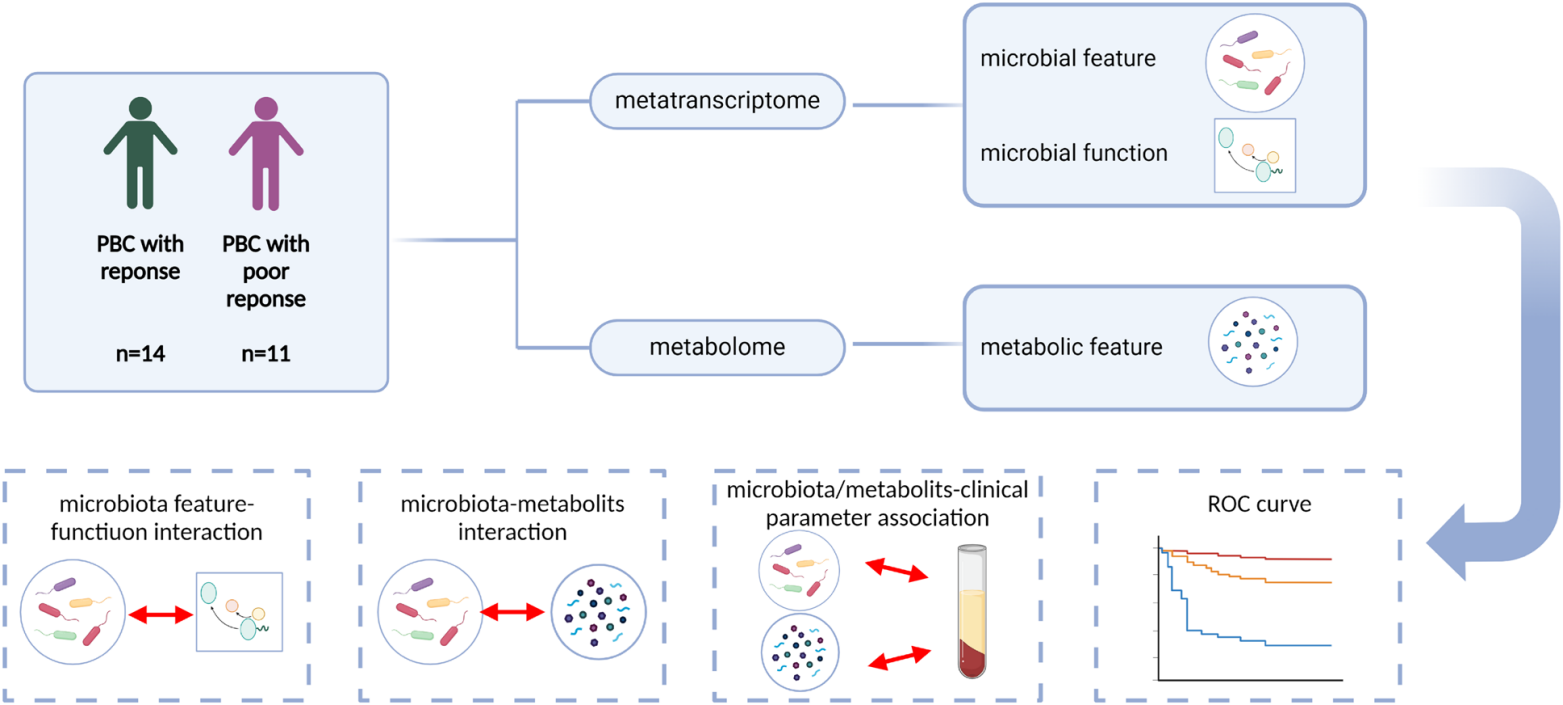
Diagram of the study design

The study design was illustrated in Fig. 1. Of 25 included patients, 14 (56%) were well response to UCDA. 11 (44%) PBC patients underwent a poor response to UCDA after 12-months treatments. Supplementary Table 1 shows the baseline characteristics for the total population and for PBC patients with and without response. The mean age was 53.42 ± 10.17 years, and most patients were female (80%). Significant differences at baseline were found for TBIL (13.06 ± 3.99 vs 41.87 ± 27.01, P = 0.005), ALT (29.36 ± 16.57 vs 67.91 ± 34.79, P = 0.005), AST (39.29 ± 24.64 vs 85.82 ± 45.42, P = 0.008), ALP (140.79 ± 71.62 vs 295.17 ± 170.04, P = 0.015), TBA (12.84 ± 12.32 vs 51.83 ± 47.13, P = 0.022). The mean Sex, Age, GGT and ALB were not significantly different between groups.

### Taxonomic changes in gut microbiome in PBC patients with poor response

**Fig. 2:**
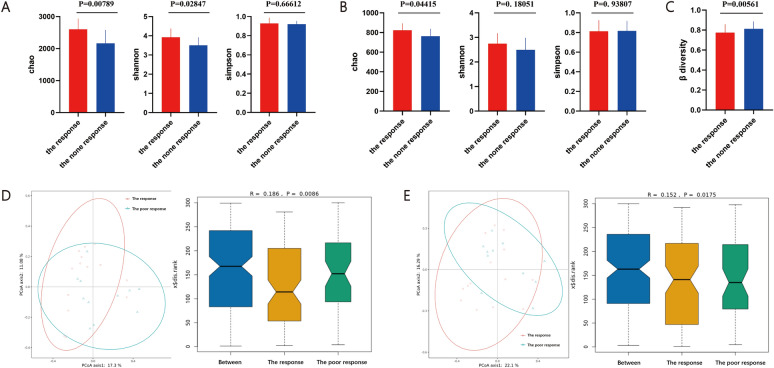
Fecal microbiome variations in PBC with poor response (the response n = 14 versus the none response n = 11 n represent biological replicates). **A** Alpha diversity comparison of two groups at the species level; **B** Alpha diversity comparison of two groups at the species level; **C** Beta diversity comparison of two groups; **D** The PCoA and NMDS overall bacterial community structure of the two groups at the genus level; **E** The PCoA and NMDS overall bacterial community structure of the two groups at the species level. *P < 0.05

First, the microbial diversity of the two groups was analyzed. For alpha diversity, significant differences were found in the Chao index and Shannon index of bacteria between the PBC patients with and without response at the species level, but no significant differences were found in the Simpson index (Fig. 2A, Supplementary Fig. 2A). At the genus level, Chao index was significant different between the two groups, while Simpson index and Shannon index showed no significant differences (Fig. 2B, Supplementary Fig. 2B). To further identify the overall microbial features of the two groups, beta diversity comparison and PCoA based on Bray–Curtis distance were performed. The results showed that the overall bacterial community structure of PBC with poor response was significantly different from PBC with response at the species level (Anosim test, P = 0.0086) and genus level (Anosim test, P = 0.0175) (Fig. 2C–E, Supplementary Fig. 2C). These results suggested that the taxonomic diversity with respect to its richness and evenness were not significant different between the two groups.

**Fig. 3:**
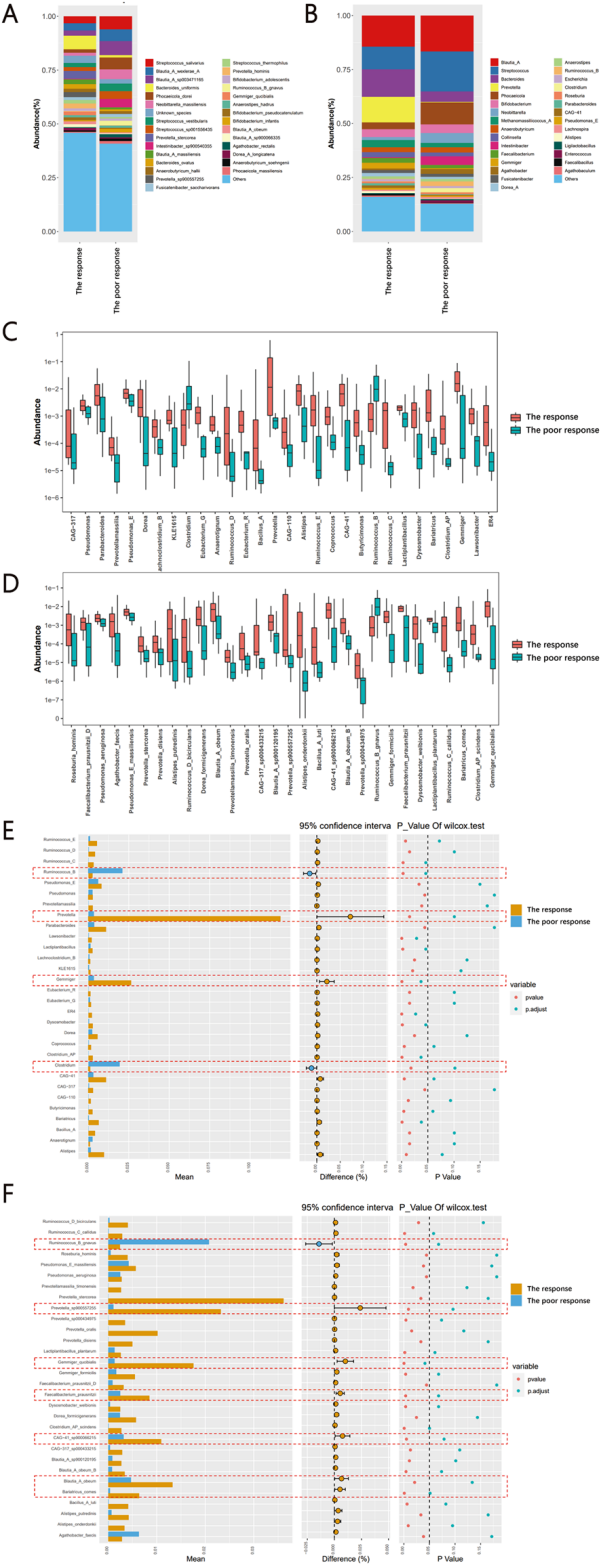
Gut microbiota signatures in PBC with poor response (the response n = 14 versus the none response n = 11, n represent biological replicates). **A** The composition of microbiota in the two group at the genus level; **B** The composition of microbiota in the two group at the species level; **C** Boxplots show the relative abundance of taxa exclusively altered in PBC with poor response at the genus level; **D** Boxplots show the relative abundance of taxa exclusively altered in PBC with poor response at the species level; **E** Stampplot show the relative abundance of taxa exclusively altered in PBC with poor response at the genus level; **F** Stampplot show the relative abundance of taxa exclusively altered in PBC with poor response at the species level

Next, the microbial composition at different taxonomic levels was analyzed. The microbial composition at the species and genus level was shown in Fig. 3A and B. 30 taxa showed the most significantly differentially altered at the genus level and 30 taxa showed the most significantly differentially altered at the species level in the poor response group (Fig. 3C and D, Supplementary Tables 2, 3). Microbiota belongs to *Gemmiger*, *Prevotella*, *Ruminococcus_B* and *Clostridium* genera showed significantly different between the two groups (Fig. 3E), and *Gemmiger_qucibialis*, *Bariatricus_comes*, *Faecalibacterium_prausnitzii*, *Blautia_A_obeum*, *CAG-41_sp900066215* and *Prevotella_sp900557255* were significantly decreased, and *Ruminococcus_B_gnavus* was significantly increased in the poor response group at the species level (Fig. 3F). The differential microbiota showed in the Fig. 3F were the differential microbiota for the further research.

### Changes in microbial function in PBC patients with poor response

**Fig. 4:**
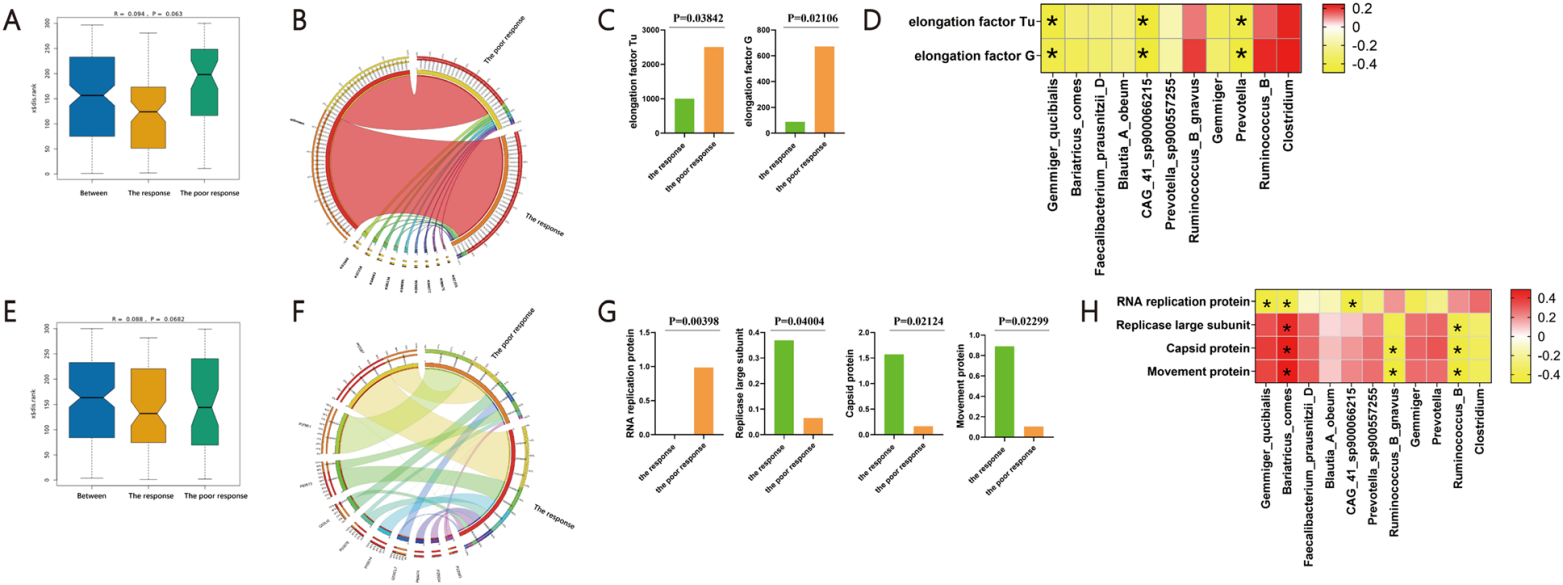
Microbiota function in PBC with poor response (the response n = 14 versus the none response n = 11, n represent biological replicates). **A** The overall microbiota transcript function of the two groups based on the KO database; **B** The overall microbiota function community structure of the two groups based on the KO database; **C** The significantly different microbiota function between the group based on the KO database; **D** Spearman analysis showed the relationship between differential microbiota and microbiota function based on the KO database; **E** The overall microbiota transcript function of the two groups based on the swissprot database; **F** The overall microbiota function community structure of the two groups based on the swissprot database; **G** The significantly different microbiota function between the group based on the swissprot database; H. Spearman analysis showed the relationship between differential microbiota and microbiota function based on the swissprot database. * < 0.05

First, we assessed the microbial function annotated by the KO database. According to the KO database, the overall microbiota transcript function of the poor response group was not significantly different from the response group (Anosim test, P = 0.063) (Fig. 4A). There were 9 functions, including enolase [EC:4.2.1.11], elongation factor Tu, molecular chaperone DnaK, glyceraldehyde 3-phosphate dehydrogenase (phosphorylating) [EC:1.2.1.12], ATP-dependent Clp protease ATP-binding subunit ClpL, formate C-acetyltransferase [EC:2.3.1.54], chaperonin GroEL [EC:5.6.1.7], glucose-1-phosphate adenylyltransferase [EC:2.7.7.27] andelongation factor G, showed mainly absolute abundance in both the two group (Fig. 4B, Table 1). Among these 9 functions, elongation factor Tu and elongation factor G were significantly increased in the poor response group (Fig. 4C, Supplementary Table 4, Supplementary Fig. 3A). To further elucidate the association between the microbiota and microbiota function, spearman analysis was carried out. *Gemmiger_qucibialis*, *CAG_41_sp900066215* and *Prevotella* showed the significant negatively relationship with both elongation factor Tu and elongation factor G (Fig. 4D, Supplementary Table 5). These results suggested the effect of bacterial function to the host.

**Table 1:** The mainly absolute abundance in both the two group. based on the KEGG database (https://www.kegg.jp/kegg/kegg2.html)

ID	Name	Pathway	Disease	Module
K01689	Enolase [EC:4.2.1.11]	map00010; map00680; map01100; map01110; map01120; map01200; map01230; map03018; map04066	H00069; H01762; H01953	M00001; M00002; M00003; M00346
K02358	Elongation factor Tu	map04626	H00891	
K04043	Molecular chaperone DnaK	map03018; map04212; map05152	H00982; H02343	
K00134	Glyceraldehyde 3-phosphate dehydrogenase (phosphorylating) [EC:1.2.1.12]	map00010; map00710; map01100; map01110; map01120; map01200; map01230; map04066; map05010; map05130; map05132; map05415		M00001; M00002; M00003; M00165; M00308; M00552
K04086	ATP-dependent Clp protease ATP-binding subunit ClpL			
K00656	Formate C-acetyltransferase [EC:2.3.1.54]	map00620; map00640; map00650; map01100; map01120		
K04077	Chaperonin GroEL [EC:5.6.1.7]	map03018; map04212; map04940; map05134; map05152; map05417	H00266; H00679	
K00975	Glucose-1-phosphate adenylyltransferase [EC:2.7.7.27]	map00500; map00520; map01100; map01110; map01250; map02026		M00565; M00854
K02355	Elongation factor G		H00891	

Then, we assessed the microbial function annotated by the SWISS-PROT database. According to the SWISS-PROT database, the overall microbiota transcript function of the poor response was also not significantly different from the response group (Anosim test, P = 0.0682) (Fig. 4E). There were 10 proteins, including RNA replication protein, Movement protein, Enolase, Capsid protein, Replicase large subunit, Probable ATP-dependent Clp protease ATP-binding subunit, Capsid protein, Movement protein and Pyruvate, phosphate dikinase, showed mainly absolute abundance in both the two group (Fig. 4F, Table 2). Among these 10 proteins, RNA replication protein was significantly decreased, while the Replicase large subunit, Capsid protein and Movement protein were significantly increased in the poor response group (Fig. 4G, Supplementary Table 6, Supplementary Fig. 3B). Spearman analysis was carried out to elucidate the association between the microbiota and microbiota function. *Bariatricus_comes* showed the significantly positively relationship with Replicase large subunit, Capsid protein and Movement protein, while significantly negatively related to RNA replication protein. *Ruminococcus_B* showed the significant negatively relationship with Replicase large subunit, Capsid protein and Movement protein. RNA replication protein showed the significant negatively relationship with *Gemmiger_qucibialis* and *CAG_41_sp900066215* (Fig. 4H, Supplementary Table 7). These results suggested the bacterial function effected by the host gut environment.

**Table 2:** The mainly absolute abundance in both the two group. based on the swissprot database (https://www.uniprot.org/)

Uniprot ID	Name	GO annotations
P20951	RNA replication protein	MF: ATP binding Source; ATP hydrolysis activity; mRNA methyltransferase activity; RNA binding; RNA helicase activity; RNA-dependent RNA polymerase activity BP: DNA-templated transcription; RNA processing; viral RNA genome replication
P69513	Movement protein	CC: host cell cytoplasm; host cell plasmodesma; host cytoskeleton; viral replication complex MF: RNA binding; BP: symbiont-mediated perturbation of host defense-related programmed cell death; transport of virus in host, cell to cell
Q03LI0	Enolase	CC: cell surface; extracellular region; peptidoglycan-based cell wall; phosphopyruvate hydratase complex MF: magnesium ion binding; phosphopyruvate hydratase activity BP: glycolytic process
P03576	Capsid protein	CC: helical viral capsid Molecular Function structural molecule activity
P69514	Replicase large subunit	MF: ATP binding; ATP hydrolysis activity; mRNA methyltransferase; RNA binding; RNA helicase activity; RNA-dependent RNA polymerase activity; BP: DNA-templated transcription; RNA processing; viral RNA genome replication;
Q5XCL7	Probable ATP-dependent Clp protease ATP-binding subunit	MF: ATP binding; ATP hydrolysis activity
P69474	Capsid protein	CC: helical viral capsid MF: structural molecule activity
P25034	Movement protein	CC: host cell cytoplasm; host cell plasmodesma; host cytoskeleton MF: RNA binding BP: transport of virus in host, cell to cell
P22983	Pyruvate, phosphate dikinase	MF: ATP binding; kinase activity; metal ion binding; pyruvate, phosphate dikinase activity BP: phosphorylation; pyruvate metabolic process

*MF* molecular function, *BP* biological process, *CC* cellular component

Microbiota derived bile salt hydrolase (BSH) was identified as one of the most priority enzyme in BAs metabolism [10]. So, we also assessed the level of the BSH in the two groups annotated by the KO database. While there was no significant difference in the abundance of BSH between the two group (Supplementary Fig. 4A). The different phylotypes of microbiota derived BSH was retrieved in the UniProt database (https://www.uniprot.org/). BSH derived from 9 kinds of *Lactobacillus* genera, 4 kind of *Bifidobacterium* genera and *Blautia_obeum* were detected (Supplementary Table 8). The B*lautia_obeum*, which was the significantly decreased species in the poor response group mentioned above, producing lower level of BSH (uniport ID A0A174NYZ7) (P = 0.048) in the poor response group. In addition, The *Lactobacillus_plantarum* (uniport ID M1R991) (P = 0.034) derived BSH was significant lower, while the gene of *Lactobacillus_salivarius* (uniport ID C7AQY2) (P = 0.045) and *Bifidobacterium_longum* (uniport ID Q9KK62) (P = 0.009) derived BSH were significant higher in the poor response group (Supplementary Fig. 4B, Supplementary Table 8). These results suggested that the level of BSH may not affect the prognosis the PBC.

Taken together, these results indicate notable changes in the microbial function in PBC patients with poor response, which may contribute to disease development.

### Associations of fecal metabolites in PBC patients with poor response

**Fig. 5:**
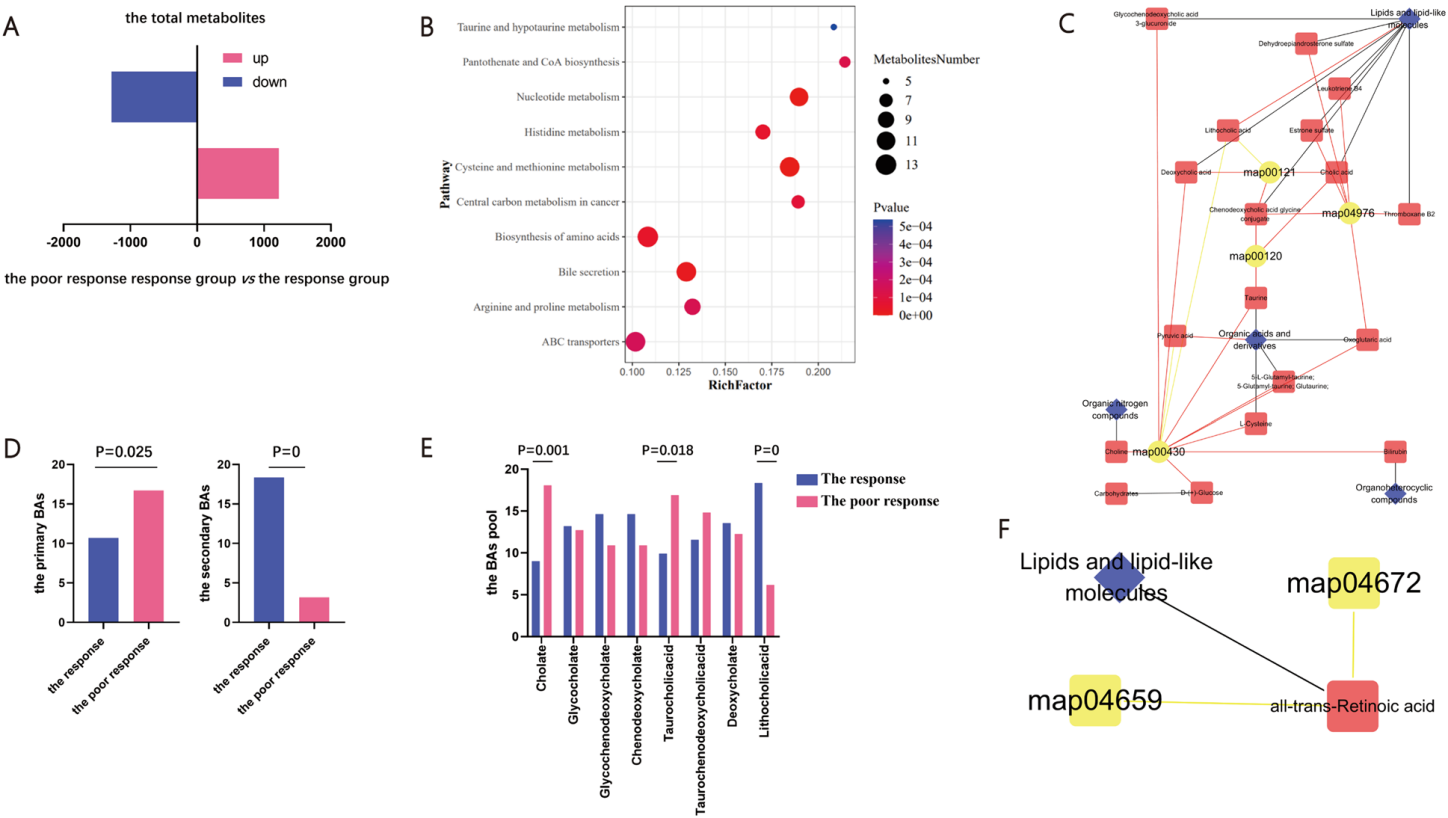
The fecal metabolite in PBC with poor response (the response n = 14 versus the none response n = 11, n represent biological replicates). **A** The number of differential metabolites in the poor response group compared to the response group; **B** The bubble chart of metabolites pathway between the two group. The X-axis Rich Factor is the number of differential metabolites annotated in this Pathway divided by all identified metabolites annotated in this Pathway. The higher the value is, the higher the ratio of differential metabolites annotated in this Pathway is. The dot size represents the number of differential metabolites annotated in this Pathway; **C** BAs metabolism related pathway enrichment analysis network plot. The circles represent metabolic pathway, and the triangles represent metabolites, and rhombus represent the class of the metabolites. Red indicates up-regulation and yellow indicates down-regulation; **D** The rank sum test showed the primary BAs and secondary BAs in the two groups; **E** The rank sum test showed the BAs pool in the two groups; **F** Immune related pathway enrichment analysis network plot. The circles represent metabolic pathway, and the triangles represent metabolites, and rhombus represent the class of the metabolites. Yellow indicates down-regulation

To further elucidate the alteration of bacterial metabolism, we performed untargeted metabolomics using the fecal samples. Compared with the response group, there was significant overall distribution of metabolite in the poor response group according to the orthogonal partial least squares discriminant analysis (OPLS-DA) (Supplementary Fig. 5A). In order to judge the quality of the model without fitting risk, 200 response permutation tests were performed on the OPLS-DA model (Supplementary Fig. 5B). Based on the differential metabolites screening criteria: (1) VIP ≥ 1; (2) Fold Change ≥ 1.2 or ≤ 0.83; (3) p-value < 0.05. There was 1223 up regulated metabolites and 1281 down regulated metabolites in the poor response group compared to the response group. By screening BMDB, HMDB, KEGG, lipidmaps, Masslist and mzCloud database, we screened out 182 metabolites that were critical for host physiological progress. The results showed significant decreased pathway in Nucleotide metabolism, Bile secretion and Histidine metabolism between the two groups. (Fig. 5A, B, Supplementary Table 9). These results suggested that the multiple differential metabolites between the two groups.

Dysbiosis of BAs metabolism is one of the main features of PBC. Then, we assessed the differential metabolites related to BAs metabolism. There were 17 differential metabolites relating to the altered BAs metabolism in PBC with poor response, including L-Cysteine, Pyruvic acid, [5-L-Glutamyl-taurine 5-Glutamyl-taurine Glutaurine], Leukotriene B4, Thromboxane B2, Oxoglutaric acid, Choline, D-( +)-Glucose, Glycochenodeoxycholic acid 3-glucuronide, Bilirubin, Chenodeoxycholic acid glycine conjugate, Cholic acid, Taurine, Deoxycholic acid, Lithocholic acid and Estrone sulfate. These metabolites belong to five class, including Organic acids and derivatives, Lipids and lipid-like molecules, Organic nitrogen compounds, Carbohydrates and Organoheterocyclic compounds. These metabolites effect four BAs related pathway, including map00430 (Taurine and hypotaurine metabolism), map04976 (Bile secretion), map00120 (Primary bile acid biosynthesis) and map00121 (Secondary bile acid biosynthesis). (Fig. 5C, Supplementary Figs. 7–10, Supplementary Table 9) Base on the rank sum test, we found the higher level of primary BAs (P = 0.025, Mann–Whitney U 36) and lower level of secondary BAs (P = 0, Mann–Whitney U 2) in the poor response group compared to the response group (Fig. 5D and Supplementary Fig. 6). In the BAs pool, the significant lower level of Cholate (P = 0.001, Mann–Whitney U 21) and Taurocholic acid (P = 0.018, Mann–Whitney U 57), as well as significant higher level of Lithocholic acid (P = 0, Mann–Whitney U 2) were detected in the response group (Fig. 5E and Supplementary Fig. 6). Dysbiosis of immune system is also one of the main features of PBC. Then, we assessed the differential metabolites related to abnormal immune response. The all-trans-Retinoic acid is decreased in the poor response group. The all-trans-Retinoic acid belongs to the Lipids and lipid-like molecules class and related to two pathways related to immune progression, including map04659 (Th17 cell differentiation) and map04672 (Intestinal immune network for IgA production) (Fig. 5F and Supplementary Figs. 11–12 and Supplementary Table 9). These results showed the abnormal metabolites related to BAs metabolism and immune response in PBC with poor response.

**Fig. 6:**
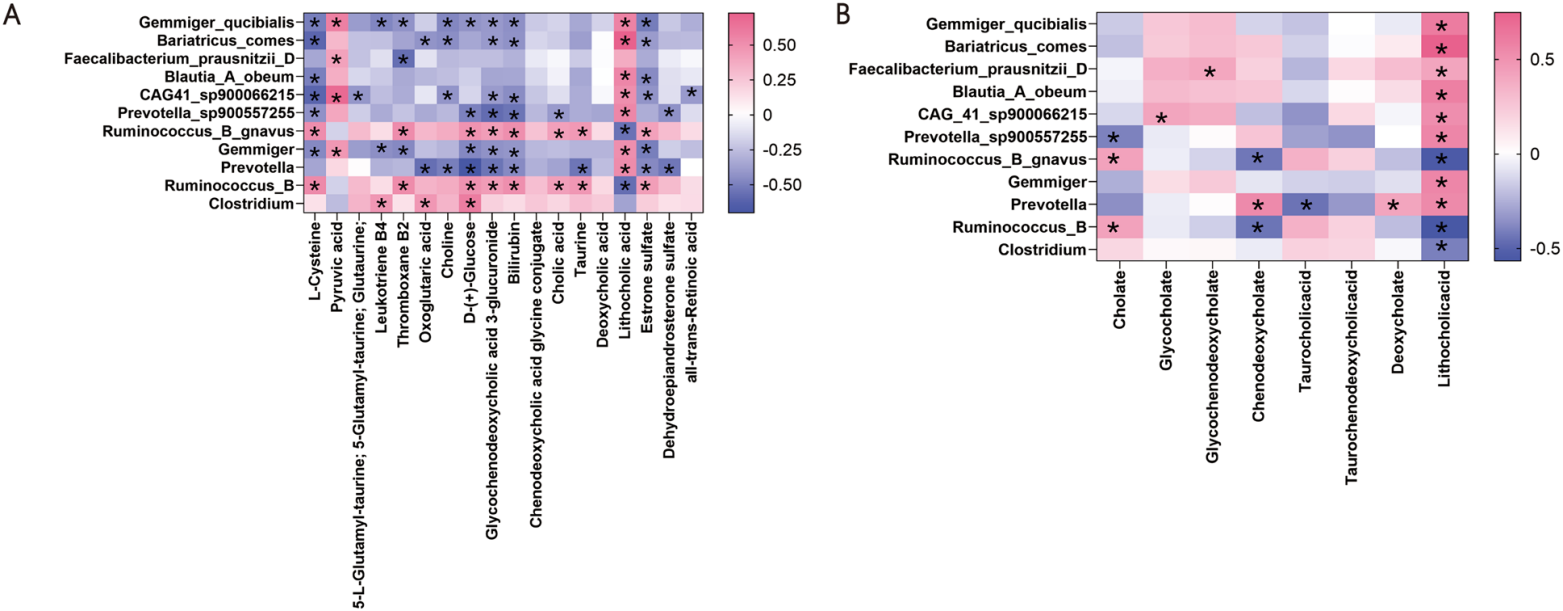
The spearman analysis showed the relationship between microbiota and metabolites (**A**), microbiota and BAs pools (**B**). * < 0.05; n = 25, n represent biological replicates

Next, we performed correlation analysis to investigate the associations between differentially abundant bacteria and metabolites. We found that bacteria enriched in the response group had strongly positive correlation with the response group-enriched metabolites but were negatively correlated with metabolites enriched in the poor response group (Fig. 6A and Supplementary Table 10). Notably, we found that some less abundant secondary BAs (e.g. Lithocholic acid) and more abundance of primary BAs (e.g. Taurocholic acid) were positively correlated with differential bacteria in the poor response group mentioned above (Fig. 6B and Supplementary Table 11).

Taken together, these results indicate the notable changes in the metabolites, the effects of altered metabolites on physiological process, and the relationship between differential expressed microbiota and metabolites in PBC with poor response, which may contribute to disease development.

### Associations of the altered microbes and metabolites with clinical parameters

**Fig. 7:**
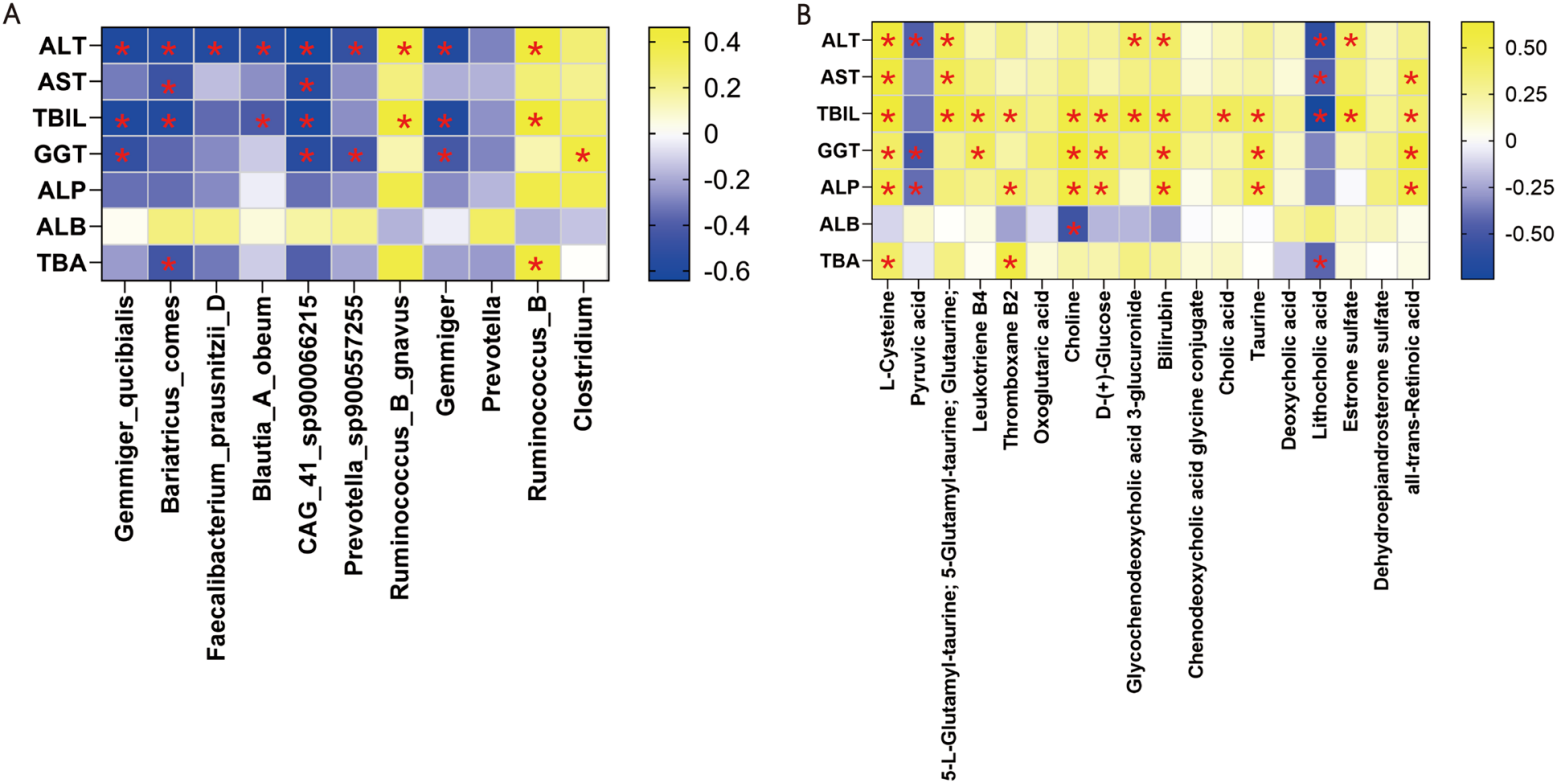
The spearman analysis showed the relationship between clinic indictors and microbiota (**A**), clinic indictors and metabolites (**B**). * < 0.05; n = 25, n represent biological replicates

Then the associations of clinical parameters and differentially abundant bacteria or metabolites were carried out. The differential expressed bacteria showed significant correlations with ALT, TBIL and total BAs, which were in accordance with previous study. For instance, *Gemmiger_qucibialis* was negatively correlated with ALT, TBIL and total BAs (Fig. 7A and Supplementary Table 12). 10 metabolites, including L-Cysteine, 5-L-Glutamyl-taurine, all-trans-Retinoic acid, Estrone sulfate, Taurine, D-( +)-Glucose, Glycochenodeoxycholic acid 3-glucuronide, Leukotriene B4 and Thromboxane B2, were positively associated with ALT, AST, TBIL, GGT, ALP and TBA. Pyruvic acid was negatively ALT, GGT and ALP (Fig. 7B and Supplementary Table 13). For instance, L-Cysteine was positively correlated with ALT, AST, TBIL, GGT, ALP and TBA. These findings suggest the effect of gut microbiota and bacterial metabolites in liver function parameters in PBC with poor prognosis, which may contribute to disease development.

### Classifier discriminating PBC with poor response from PBC with response

**Fig. 8:**
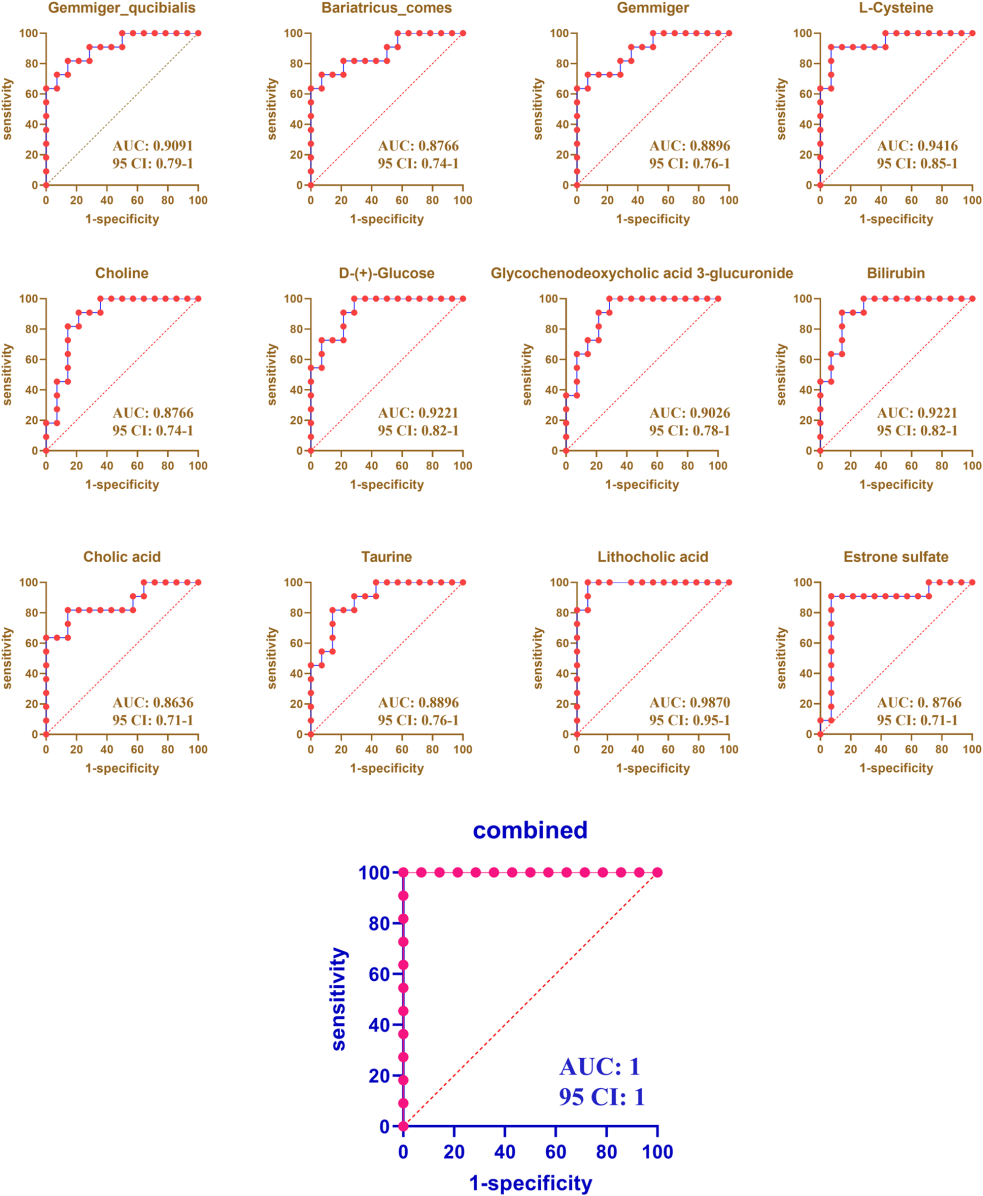
Disease classification based on the ROC plot. X-axis represents 1-specificity, y-axis represent sensitivity. The area under the curve is the AUC value. A higher AUC value indicates a more suitable metabolite as a biomarker

The value of using the differential microbiota and metabolites as biomarkers for the differential diagnosis of PBC with poor response was assessed. Through the receiver operating characteristic (ROC) curve (AUC > 0.85), we identified 1 bacterial genera, 2 bacterial species and 9 metabolites that could distinguish PBC patients with poor response from patients with response (Fig. 8).

## Discussion

In this study, we firstly used metatranscriptomics and metabolomic analysis to identify not only the altered gut bacteria but also microbial functions and metabolites in PBC patients with poor response in comparison with PBC patients with response. The fecal metatranscriptomic profiles showed the altered microbiota and microbiota function in PBC with poor response. The fecal metabolomic profiles were significantly distinct the two study groups and were tightly linked with microbiota compositions. Importantly, we found that microbial and metabolic changes in PBC patients with poor response were associated with the serum level of total BAs, aminopherase and TBIL. Then, we validated gut bacteria and metabolites that can distinguish PBC patients with poor response from patients with response. Most of the studies have been focusing on gut microbiota signature, little is known about microbial functions and metabolites in PBC, thus, our findings lay the foundation for better understanding the role of the gut microbiota in different stage of PBC.

In the current study, we identified distinctive signatures of microbiota in PBC patients with poor response. The microbiota of patients in the poor response group showed significantly decreased abundance of *Gemmiger* and *Prevotella*, as well as significantly increased abundance of *Clostridium* and *Ruminococcus* at the genus level. *Gemmiger_qucibialis*, *Bariatricus_comes*, *Faecalibacterium_prausnitzii*, *Blautia_A_obeum*, *CAG-41_sp900066215* and *Prevotella_sp900557255* were significantly decreased, and *Ruminococcus_B_gnavus* were significantly increased in the poor response group at the species level. The gut microbiota is essential in the regulation of primary and secondary BAs pathways [13]. *Gemmiger* was suggested the core functional genera associated with the significantly affected metabolites, which are involved in the biosynthesis, degradation, and elongation of BAs [14]. Increased *Prevotella* abundance is associated with augmented Th17-mediated mucosal inflammation [15]. Studies also suggested the association between *Prevotella* abundance and changed BAs pools [16]. Greater *Ruminococcus* abundance was associated with the synthesis of secondary BAs [17]. Studies also suggested the positively relationship between *Ruminococcus* and inflammation, as well as the relationship between *Clostridium* and Th17 differentiation [18]. *Clostridium* also mediated 7α-dehydroxylation activity to suppress secondary BAs biosynthesis [19]. These results suggests that in PBC patients with poor response, changed gut microbiota is associated with promotion of Th17-mediated immune responses as well as disturbed enterohepatic circulation of BAs metabolism.

Next, we identified distinctive signatures of microbial functions in PBC patients with poor response. The microbial functions showed significantly decreased abundance of elongation factor Tu and elongation factor G base on the KO database. Elongation factor Tu is a G protein, possessing an intrinsic ability to hydrolyze GTP to GDP and contributes to the overall fidelity of translation. Elongation factor Tu plays an important role in delivering subsequent amino acyl-tRNAs to the A-site of the programmed ribosome in order to elongate the newly synthesized protein [20]. Studies had suggested that the decreased Elongation factor Tu may reduce hepatocytic triglyceride deposition, thereby decreasing steatosis, as demonstrated by the modulatory effects on ornithine aminotransferase, mitochondrial aspartate aminotransferase, acyl-CoA synthase, hydroxyacyl-CoA dehydrogenase and D-beta-hydroxybutyrate dehydrogenase [21]. Elongation factor G is a guanosine triphosphatase, binds to the ribosomal PRE-translocation complex and facilitates movement of transfer RNAs and messenger RNA by one codon [22]. Studies demonstrated that elongation factor G may promote the progression of hepatocellular carcinoma via activating signaling pathways [23]. The differential expressed microbiota in PBC with poor response, including *Gemmiger_qucibialis*, *CAG_41_sp900066215* and *Prevotella* were significant negatively with these two microbiota functions. These results suggested the promotion of host immune response by the altered microbiota. Base on the SWISS-PROT database, we found a significantly decreased abundance of Replicase large subunit, Capsid protein and Movement protein, as well as increased RNA replication protein in PBC with poor response. Replicase large subunit is kind of key RNA-synthesizing enzymes play an important role in replicase functions, including ATP binding, ATP hydrolysis activity, mRNA methyltransferase activity etc. (https://www.uniprot.org/) Capsid protein and Movement protein are the important composition of the virus. Capsid proteins of some eukaryotic viruses are known to stimulate innate immune signaling in mammalian hosts as well as constant onslaught to bacteria [24]. And Movement protein could help the virus move between cells in the host. In this research, we found a significant relationship between the altered proteins and decreased level of *Gemmiger_qucibialis*, *Bariatricus_comes*, *CAG_41_sp900066215* and *Ruminococcus*. These results suggested that the altered microbiota may be due to increased attack of virus on microbiota in PBC with poor response. Besides, Microbiota derived BSH (EC 3.5.1.24) is a member of the choloylglycine hydrolase family. Microbiota derived BSH was reported to prior carry out BA deconjugation and prevent the reabsorption of BAs into the enterohepatic circulation. BSH homologues and the protein clusters of BSH are not the same in different ethnic groups. BSH derived from different microbiota have differences in protein sequence composition, classification, structure and function. The presence of paralogs may cause differences in the functions of BSHs in the same bacteria strain [25, 26]. In this research, we observed no significant difference in total BSH between PBC patients with or without response. Then, we acquired 14 BSH derived from 3 genera and 9 species. *Blautia_obeum* derived BSH, which was reported to hydrolyzed TCA [27], was significant lower resulted from the decreased *Blautia_obeum* in the poor response group. These suggested that reduced differential microbiota derived BSH does not affect the total level of BSH in the PBC with poor response. BSH may not be a factor affecting the enterohepatic circulation of BAs in PBC.

Then, we identified distinctive signatures of gut metabolite in PBC patients with poor response. The metabolites of PBC with poor response showed significantly increased abundance of L-Cysteine, 5-L-Glutamyl-taurine, Leukotriene B4, Thromboxane B2, Oxoglutaric acid, D-( +)-Glucose, Estrone sulfate and Dehydroepiandrosterone sulfate. L-Cysteine and 5-L-Glutamyl-taurine are intermediates of taurine, which is able to increase the solubility of primary BAs. Xu et al. found that the elevated level of 5-L-Glutamyl-taurine was closely related with drug related disorder of bile acid metabolism and could be used as a marker of liver injury [28]. In this research, the elevation of these intermediate metabolites indicates increased taurine demand and increased synthesis of BAs. The level of Leukotriene B4 is in response to BAs stimulation [29]. The level of thromboxane, which relate to cirrhosis and high portal pressure, could be reduced by UDCA in the previous study [30]. Oxoglutaric acid and D-( +)-Glucose are metabolites in glucose metabolism. Estrone sulfate and Dehydroepiandrosterone sulfate are two of the most abundant steroids in the human circulation. Liu et al. suggested that the estrone sulfate level was relevant to increased BA level [31]. Previous studies had suggested the positively relationship between BAs efflux transporter in the hepatocytes and Dehydroepiandrosterone sulfate [32]. In this research, these metabolites were identified as the metabolites positively related to the bile secretion based on the KEGG database. The metabolites of PBC with poor response also showed significantly decreased abundance of Pyruvic acid. Pyruvic acid is a central metabolic intermediate in glucose metabolism. Recent study has shown that elevated levels of Pyruvic acid can alleviate acute liver injury [33]. In this research, we also found the negative relationship between Pyruvic acid and clinic liver function indicators. Furthermore, excess primary BAs cause inflammation and oxidative stress in hepatocytes. Studies have found that reduced secondary BAs and decreased secondary/primary BAs ratio are closely associated with the occurrence of end-stage liver disease in cholestatic disease [34, 35]. The primary BAs, such as taurine ursodeoxycholic acid, was reported to inhibited the activation of hepatic farnesoid X receptor, which further leads to hepatic lipid dysmetabolism [36]. Secondary BAs also could active the G protein coupled receptor, which can modulate chemical communications from the intestinal microbiota and the host's immune system, integrating epithelial cells and immune cells in the entero-hepatic system. PBC patients showed reduced transformation from primary to secondary and from conjugated to unconjugated BAs. Dysfunctional microbial metabolism leading to failure in dehydroxylation was suggested as a possible mechanism [37]. In line with those findings, we found an increased level of primary BAs and decreased level of secondary BAs in PBC with poor response. The dysbiosis of BAs enterohepatic circulation is significant with the differential expressed microbiota mentioned above, including *Prevotella_sp900557255 *etc. Studies has suggested that the level of the secondary BAs was negatively related to *Prevotella*. [38] We also found the positive relationship between the *Prevotella_sp900557255* and lithocholic acid. Besides, the lithocholic acid was positively related with *Gemmiger_qucibialis*, *Bariatricus_comes*, *Faecalibacterium_prausnitzii_D*, *Blautia_A_obeum CAG_41_sp900066215*. *Gemmiger* [14]*, Faecalibacterium* [34] and *Blautia* [39] were confirmed to be associated with the transformation of primary BAs to secondary BAs. Next, we also found a decreased level of all-trans-Retinoic acid in the PBC with poor response. Previous studies have suggested PML as auto-antigens in PBC. The all-trans-Retinoic acid is an immune inhibitor against PML [40]. In this research, the all-trans-Retinoic acid was identified as the metabolites positively related to Th17 cell differentiation and Intestinal immune network for IgA production based on the Kegg database. These results suggested that, the differential metabolites were related to dysbiosis of BAs metabolism and immune procession in PBC with poor response.

The diverse set of microbial functions may be relevant, including microbial metabolites and bacterial processing of BAs through the gut-liver axis [41, 42]. Finally, we explored the relationship between microbiota, metabolites and clinic indicators related to the liver function. The *Gemmiger *etc*.*, which decreased in the PBC with poor response, were negatively related to the transaminase, GGT and ALP. While the *Ruminococcus*, which increased in the PBC with poor response, was positively related to the total BAs, transaminase and ALP. The Pyruvic acid etc*.*, which decreased in the PBC with poor response, were negatively related to the transaminase and ALP. While the L-Cysteine etc*.*, which increased in the PBC with poor response, were positively related to the total BAs, transaminase, GGT, TBIL and ALP. Based on gut microbial signatures and metabolic features of the disease, we found the potential biomarker for the poor response of PBC, which could accurately identify the prognosis of PBC. Similar to our previous study, the *Gemmiger* genera and *Gemmiger_qucibialis* species showed the differentiation power in distinguishing PBC patients with poor response from patients with response [8]. Besides, the composition of BAs pool, including cholic acid and lithocholic acid, have the potential power in distinguishing PBC with poor response. Other metabolite, including L-Cysteine etc*.*, also showed the differentiation power in distinguishing PBC with poor response. Thus, it is possible to differentiate the disease from the perspective of gut microbiota and metabolites. At present, the methods to differentiate PBC with poor response is difficult due to unavailable facility for autoantibody detection in some clinics.

Our study has several limitations. First, our findings could be influenced by other confounders including described differences such as sex, BMI, race/ethnicity, and diet. While considerable effort was made to account for these covariates, we cannot exclude residual effects of confounding. Second, this study had a cross-sectional design, which may provide a less accurate assessment of complex microbial compositional and functional relationships compared to longitudinal studies. Third, our study only evaluated the prediction value of microbial and metabolic features, and a classification model based on the k-fold cross-validation or the Leave-One-Out is required to achieve higher efficacy and accuracy. Finally, the sample size of this study was relatively small, and the subjects were restricted to a specific ethnic population and geographic region, which may limit the generalizability of the results. The efficacy and accuracy need to be identified.

In summary, microbiota dysbiosis was identified in PBC patients with poor response. The microbiota dysbiosis related to host immune dysfunction, abnormal BAs metabolism and abnormal liver function. Our study indicates that microbiota-microbial functions-metabolite interactions are likely involved in the pathogenesis of PBC with poor response. Our study provides some insight into gut microbiota and metabolite signatures that could be useful in the identifying and treating of PBC with poor response. However, given the potential inaccuracies of gene abundance prediction, further studies incorporating shotgun metagenomics are required to confirm the findings of gene-normalized transcriptional analyses.

### Supplementary Information

**Supplementary Material 1.** Flow diagram of patient enrollment.

**Supplementary Material 2.** The violin plots showed fecal microbiome variations in PBC with poor response (the response n = 14 versus the none response n = 11, n represent biological replicates). A. Alpha diversity comparison of two groups at the species level; B. Alpha diversity comparison of two groups at the species level; C. Beta diversity comparison of two groups

**Supplementary Material 3.** The violin plots showed microbiota function in PBC with poor response (the response n = 14 versus the none response n = 11, n represent biological replicates). A. The significantly different microbiota function between the group based on the KO database; B. The significantly different microbiota function between the group based on the swissprot database

**Supplementary Material 4.** The level of total BSH and differential microbiota derived BSH in PBC with poor response (the response n = 14 versus the none response n = 11, n represent biological replicates). Q1WR93: Lactobacillus salivarius derived BSH; C7AQX8: Lactobacillus salivarius derived BSH; C7AQY2: Lactobacillus salivarius derived BSH; J7H3P9: Lactobacillus salivarius derived BSH; A0A0R2G1E1: Lactobacillus salivarius derived BSH; M1R991: Lactobacillus plantarum derived BSH; M1R367: Lactobacillus plantarum derived BSH; B9V405: Lactobacillus gasser derived BSH; P97038: Lactobacillus johnsonii derived BSH; Q9KK62: Bifidobacterium longum derived BSH; Q6R974: Bifidobacterium bifidum derived BSH; Q53CP8: Bifidobacterium animalis derived BSH; A7A5K1: Bifidobacterium adolescentis L2-32 derived BSH; A0A174NYZ7: Blautia obeum derived BSH.

**Supplementary Material 5.** A. Score diagram of OPLS-DA analysis model; B. Replacement test chart of OPLS-DA analysis model. The two points in the upper right corner represent R2 and Q2 of the actual model The dot on the left represents the displacement test result. Generally, Q2 obtained by displacement test needs to be less than Q2 of the model If you can see a red dotted line inclined upward, it indicates that the model is good and has not been fitted. (the response n = 14 versus the none response n = 11, n represent biological replicates).

**Supplementary Material 6.** The violin plots showed the BAs pool in the two group. (the response n = 14 versus the none response n = 11, n represent biological replicates).

**Supplementary Material 7.** KEGG Metabolic Enrichment Pathway.

**Supplementary Material 8.** KEGG Metabolic Enrichment Pathway.

**Supplementary Material 9.** KEGG Metabolic Enrichment Pathway.

**Supplementary Material 10.** KEGG Metabolic Enrichment Pathway.

**Supplementary Material 11.** KEGG Metabolic Enrichment Pathway.

**Supplementary Material 12.** KEGG Metabolic Enrichment Pathway.

**Supplementary Material 13.** Supplementary table.

**Supplementary Material 14.** Supplementary method.

## Data Availability

The datasets generated during the current study are available from the corresponding author upon reasonable request.
