# Supplementary figures and images for: Distinct signatures of gut microbiota and metabolites in primary biliary cholangitis with poor biochemical response after ursodeoxycholic acid treatment
Source: Cell Biosci. 2024 Jun 15;14:80. doi: 10.1186/s13578-024-01253-1 (PMC11180406; doi:10.1186/s13578-024-01253-1)

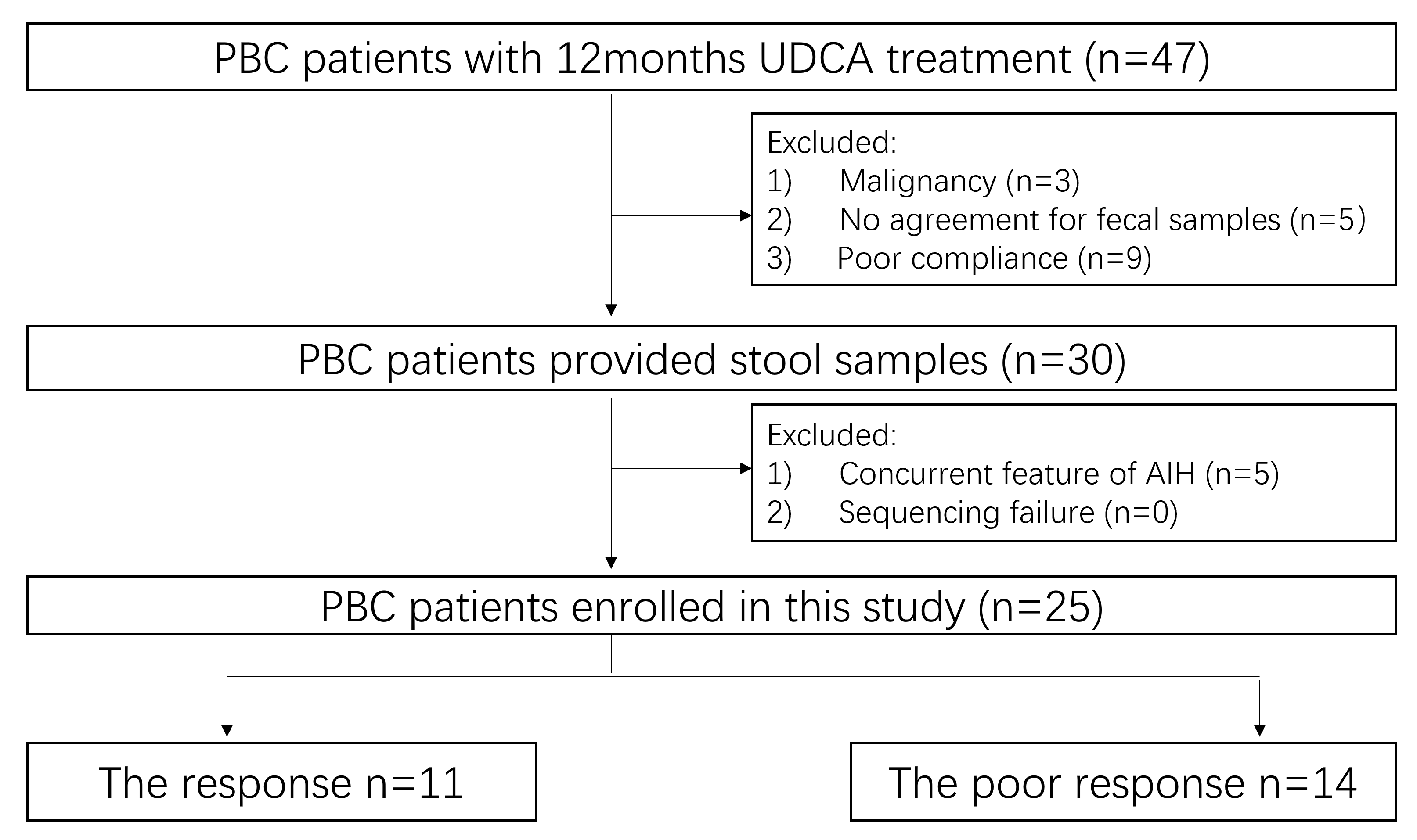

Supplement: Supplementary file 1 — Supplementary Material 1. Flow diagram of patient enrollment. [file 13578_2024_1253_MOESM1_ESM.tif]

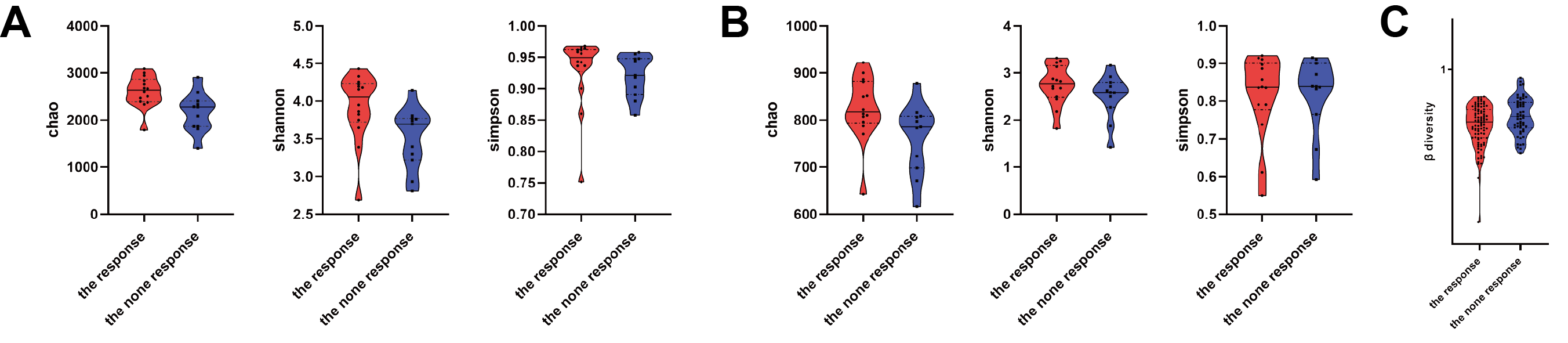

Supplement: Supplementary file 2 — Supplementary Material 2. The violin plots showed fecal microbiome variations in PBC with poor response (the response n = 14 versus the none response n = 11, n represent biological replicates). A. Alpha diversity comparison of two groups at the species level; B. Alpha diversity comparison of two groups at the species level; C. Beta diversity comparison of two groups [file 13578_2024_1253_MOESM2_ESM.png]

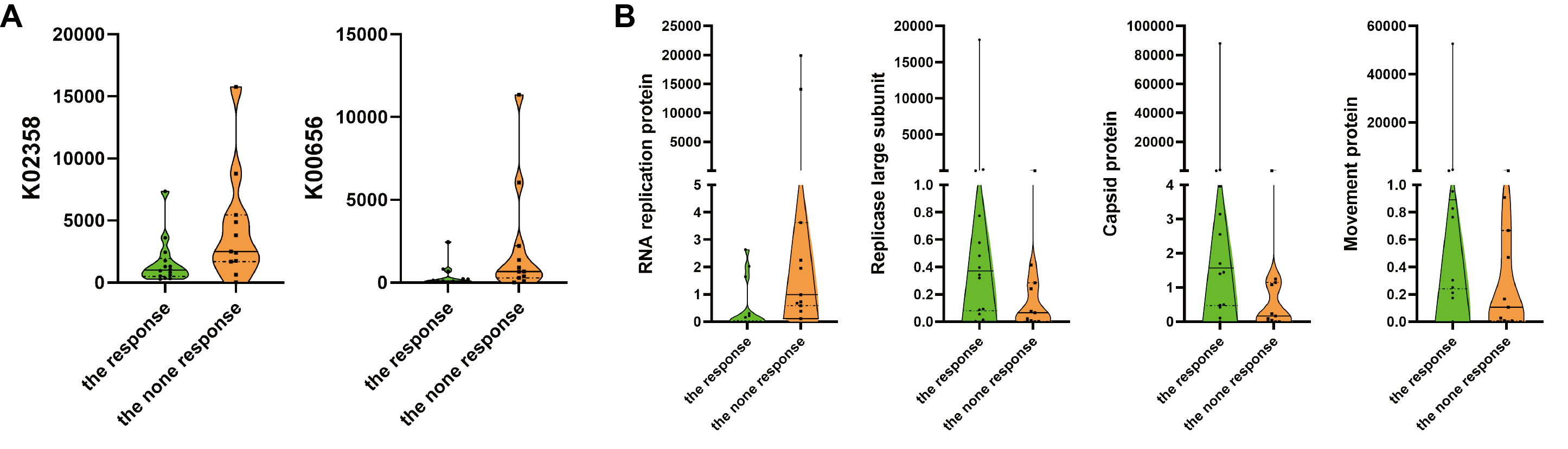

Supplement: Supplementary file 3 — Supplementary Material 3. The violin plots showed microbiota function in PBC with poor response (the response n = 14 versus the none response n = 11, n represent biological replicates). A. The significantly different microbiota function between the group based on the KO database; B. The significantly different microbiota function between the group based on the swissprot database [file 13578_2024_1253_MOESM3_ESM.png]

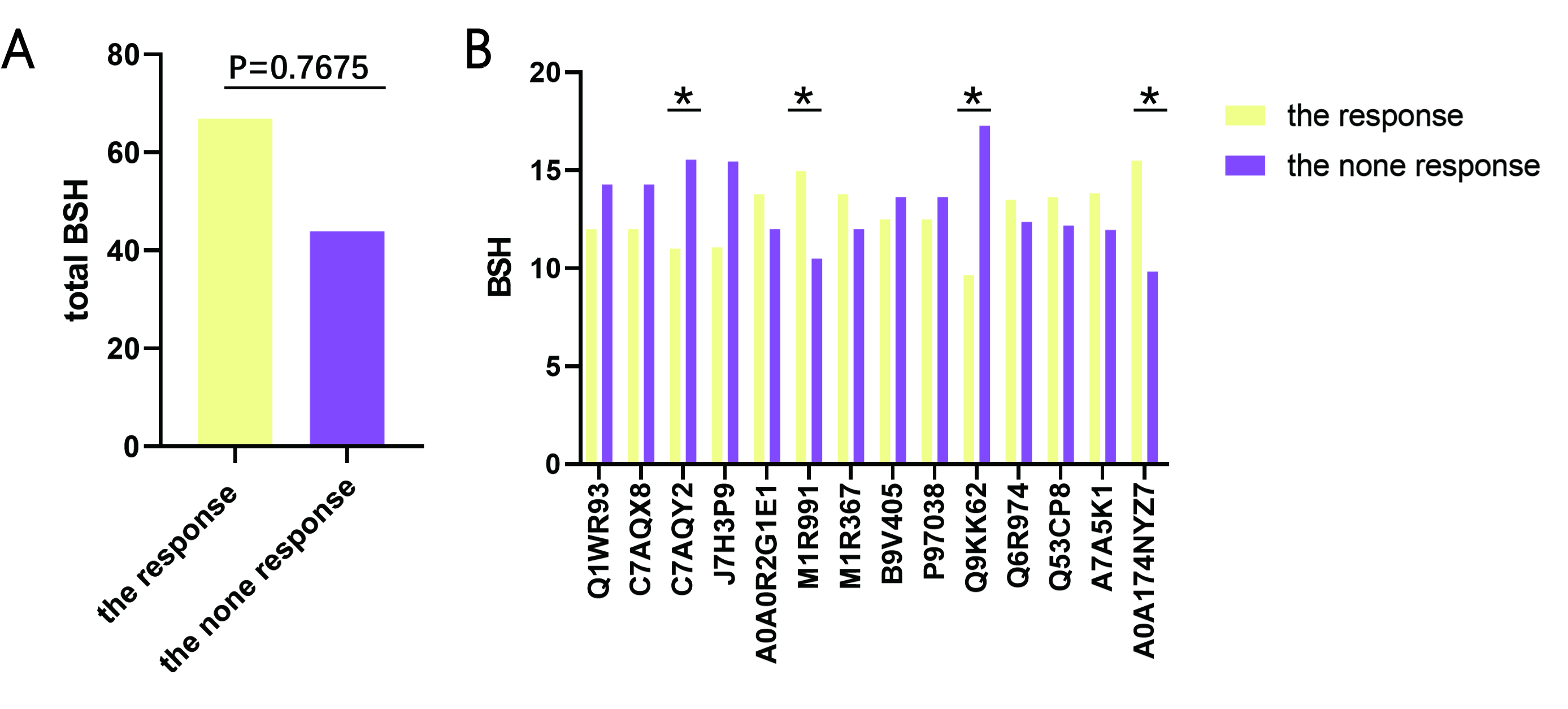

Supplement: Supplementary file 4 — Supplementary Material 4. The level of total BSH and differential microbiota derived BSH in PBC with poor response (the response n = 14 versus the none response n = 11, n represent biological replicates). Q1WR93: Lactobacillus salivarius derived BSH; C7AQX8: Lactobacillus salivarius derived BSH; C7AQY2: Lactobacillus salivarius derived BSH; J7H3P9: Lactobacillus salivarius derived BSH; A0A0R2G1E1: Lactobacillus salivarius derived BSH; M1R991: Lactobacillus plantarum derived BSH; M1R367: Lactobacillus plantarum derived BSH; B9V405: Lactobacillus gasser derived BSH; P97038: Lactobacillus johnsonii derived BSH; Q9KK62: Bifidobacterium longum derived BSH; Q6R974: Bifidobacterium bifidum derived BSH; Q53CP8: Bifidobacterium animalis derived BSH; A7A5K1: Bifidobacterium adolescentis L2-32 derived BSH; A0A174NYZ7: Blautia obeum derived BSH. [file 13578_2024_1253_MOESM4_ESM.tif]

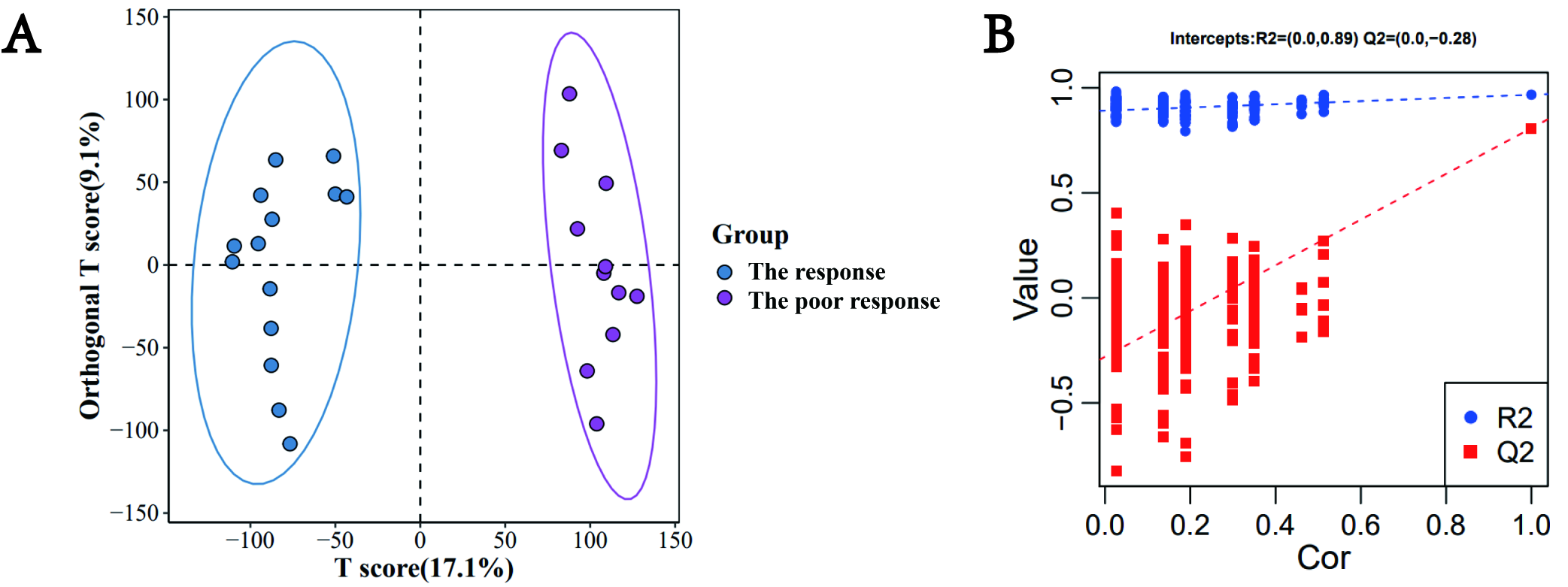

Supplement: Supplementary file 5 — Supplementary Material 5. A. Score diagram of OPLS-DA analysis model; B. Replacement test chart of OPLS-DA analysis model. The two points in the upper right corner represent R2 and Q2 of the actual model The dot on the left represents the displacement test result. Generally, Q2 obtained by displacement test needs to be less than Q2 of the model If you can see a red dotted line inclined upward, it indicates that the model is good and has not been fitted. (the response n = 14 versus the none response n = 11, n represent biological replicates). [file 13578_2024_1253_MOESM5_ESM.tif]

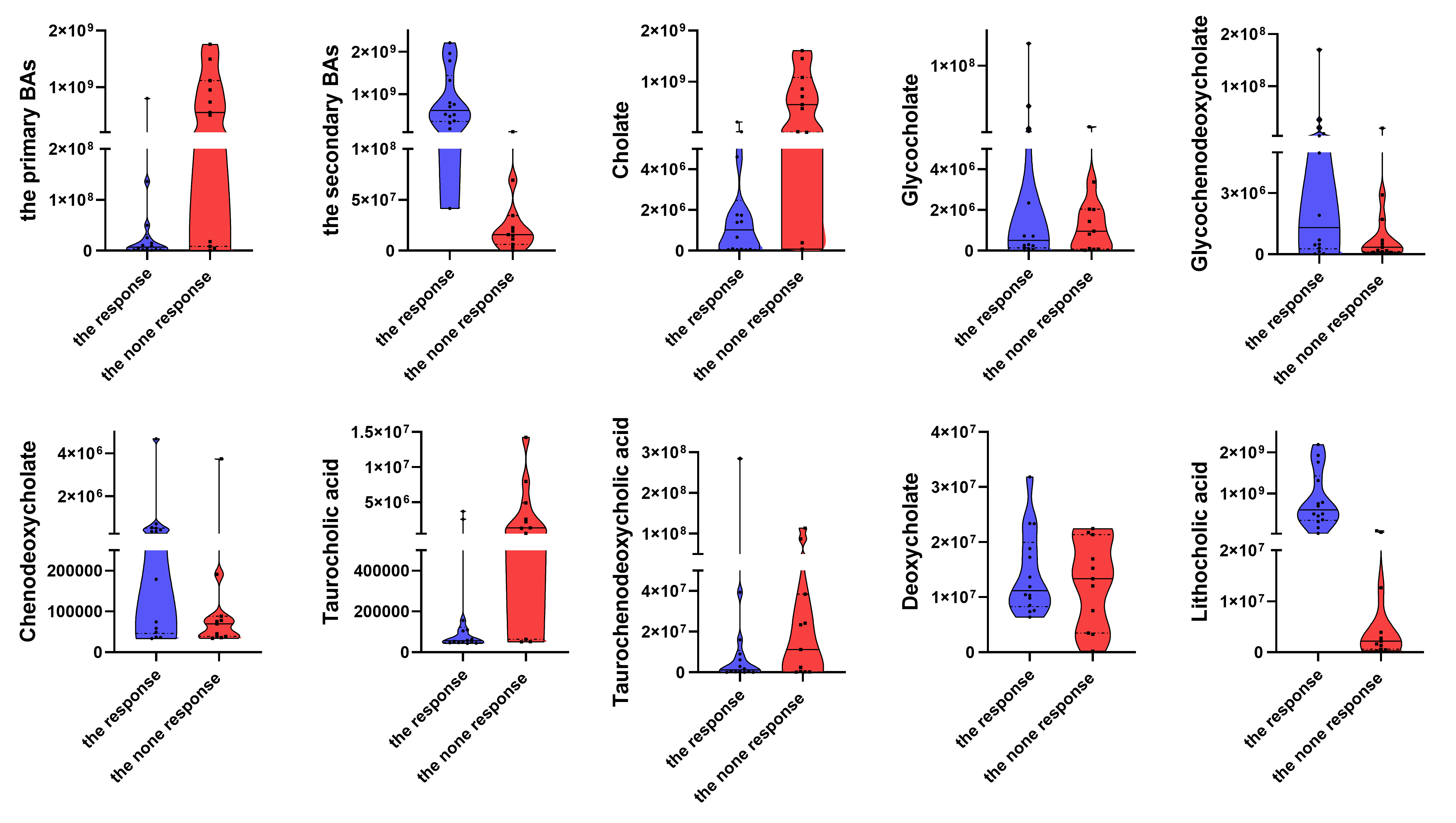

Supplement: Supplementary file 6 — Supplementary Material 6. The violin plots showed the BAs pool in the two group. (the response n = 14 versus the none response n = 11, n represent biological replicates). [file 13578_2024_1253_MOESM6_ESM.tif]

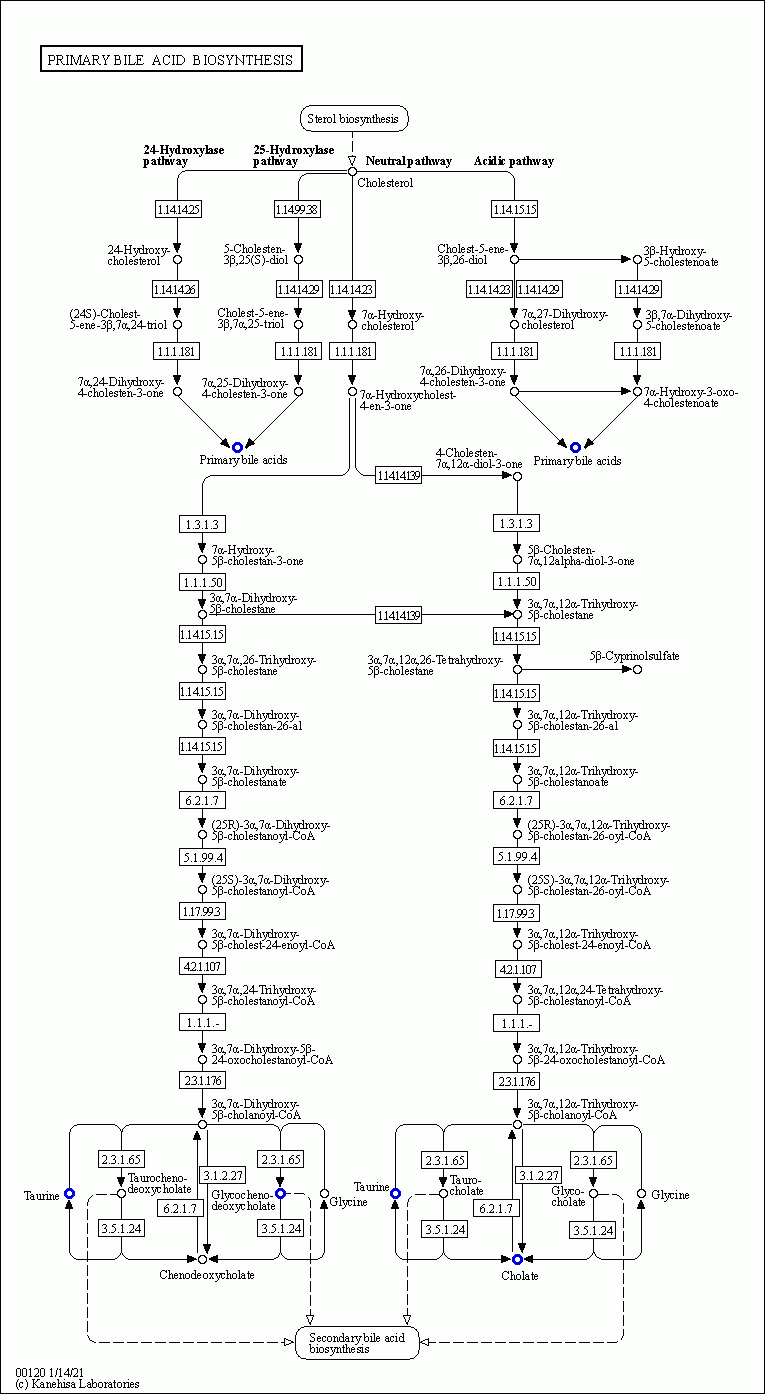

Supplement: Supplementary file 7 — Supplementary Material 7. KEGG Metabolic Enrichment Pathway. [file 13578_2024_1253_MOESM7_ESM.png]

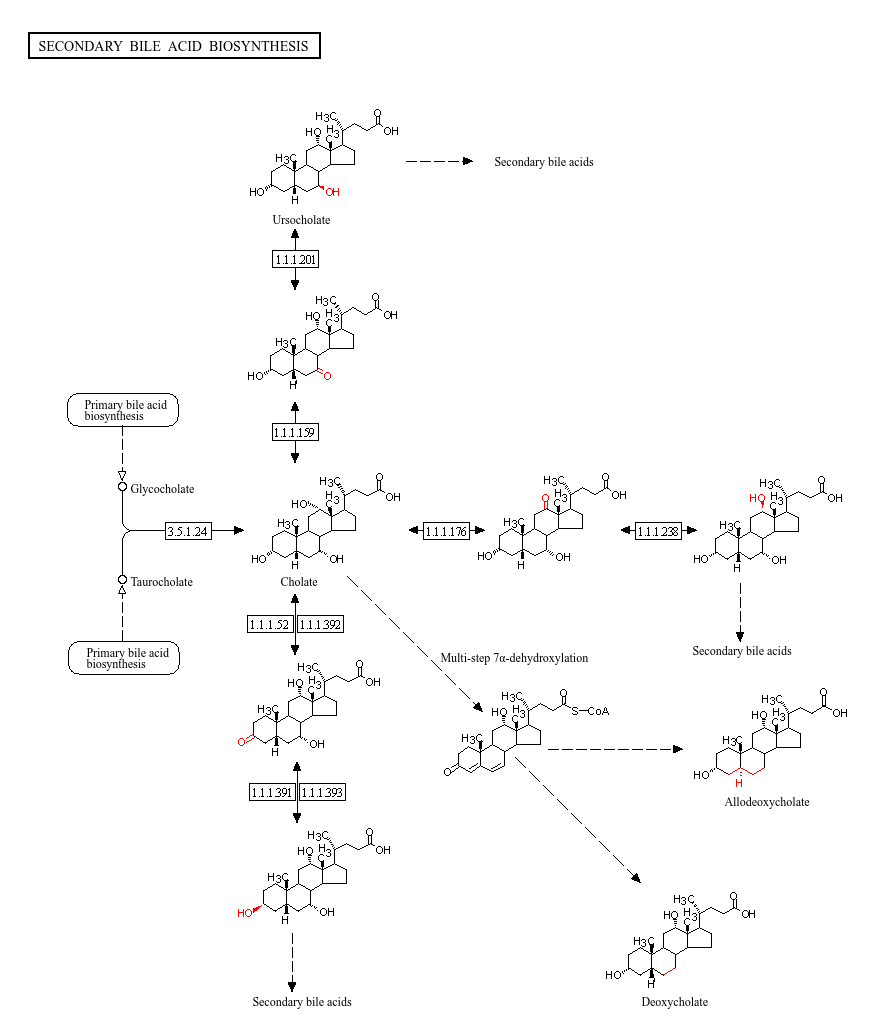

Supplement: Supplementary file 8 — Supplementary Material 8. KEGG Metabolic Enrichment Pathway. [file 13578_2024_1253_MOESM8_ESM.png]

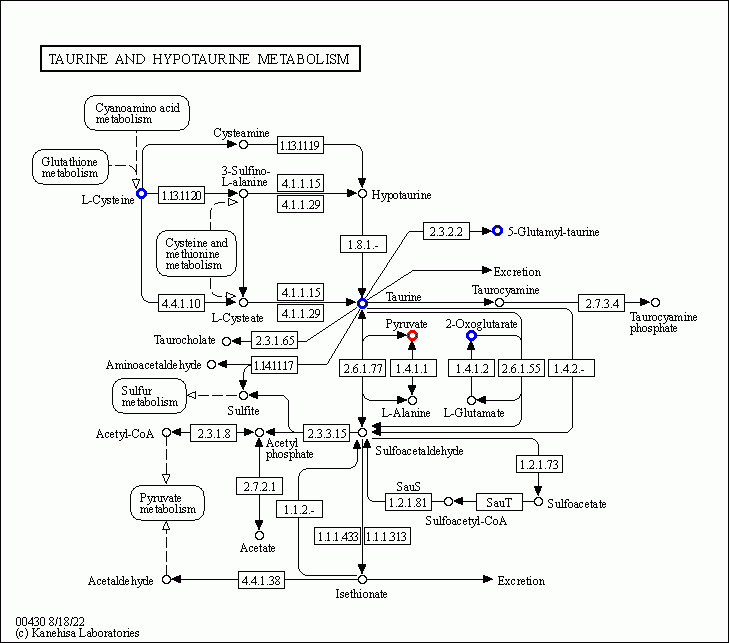

Supplement: Supplementary file 9 — Supplementary Material 9. KEGG Metabolic Enrichment Pathway. [file 13578_2024_1253_MOESM9_ESM.png]

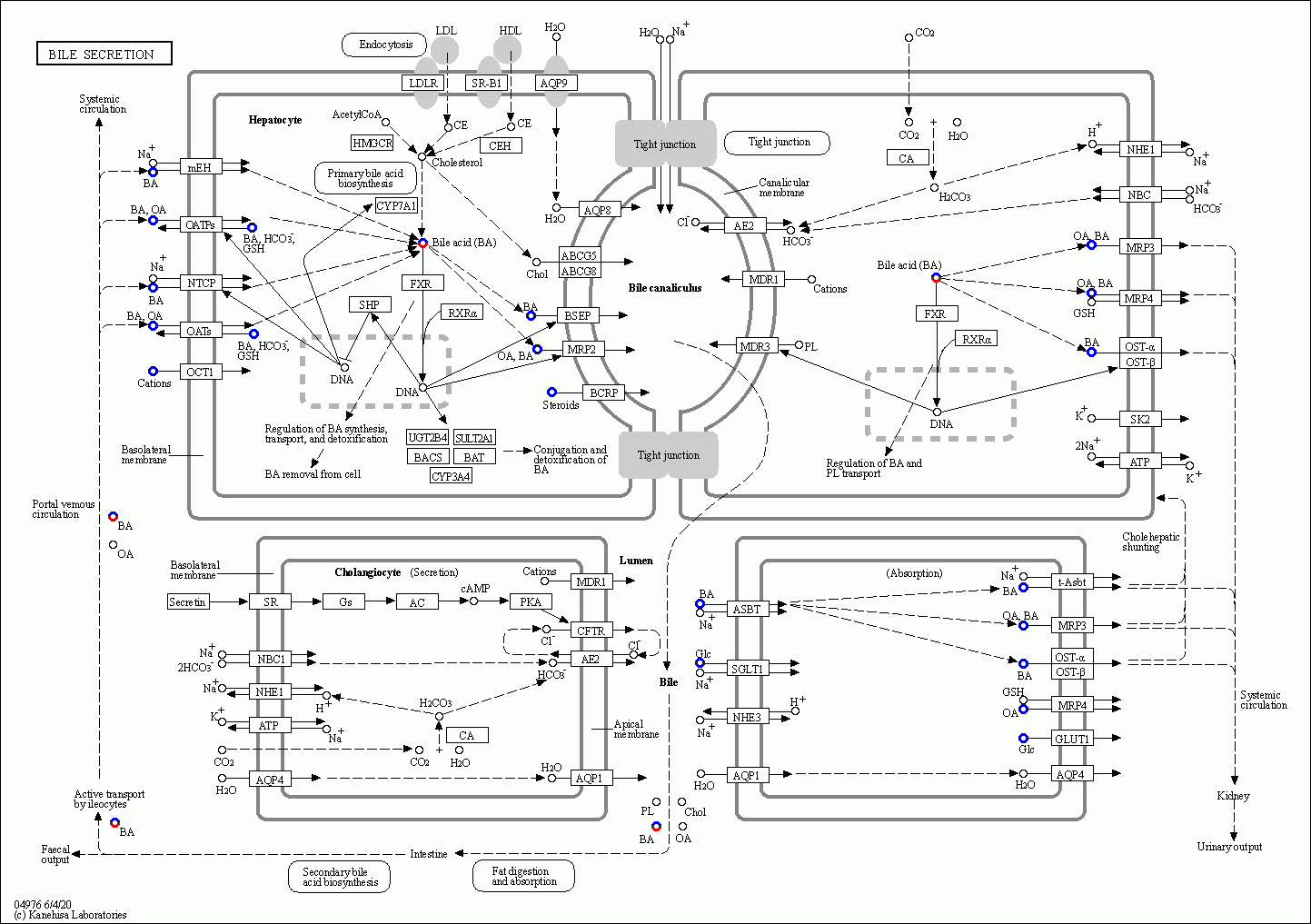

Supplement: Supplementary file 10 — Supplementary Material 10. KEGG Metabolic Enrichment Pathway. [file 13578_2024_1253_MOESM10_ESM.png]

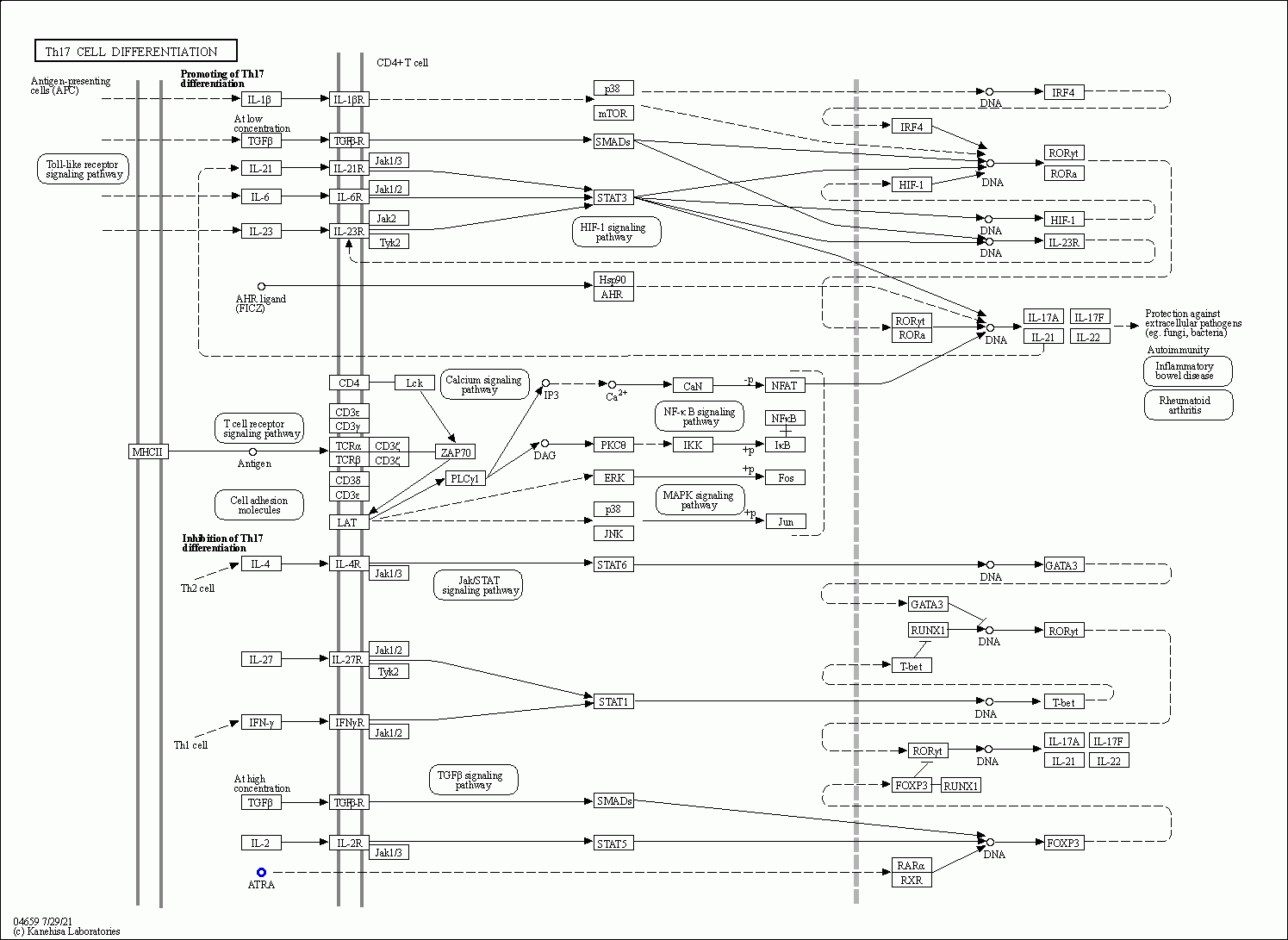

Supplement: Supplementary file 11 — Supplementary Material 11. KEGG Metabolic Enrichment Pathway. [file 13578_2024_1253_MOESM11_ESM.png]

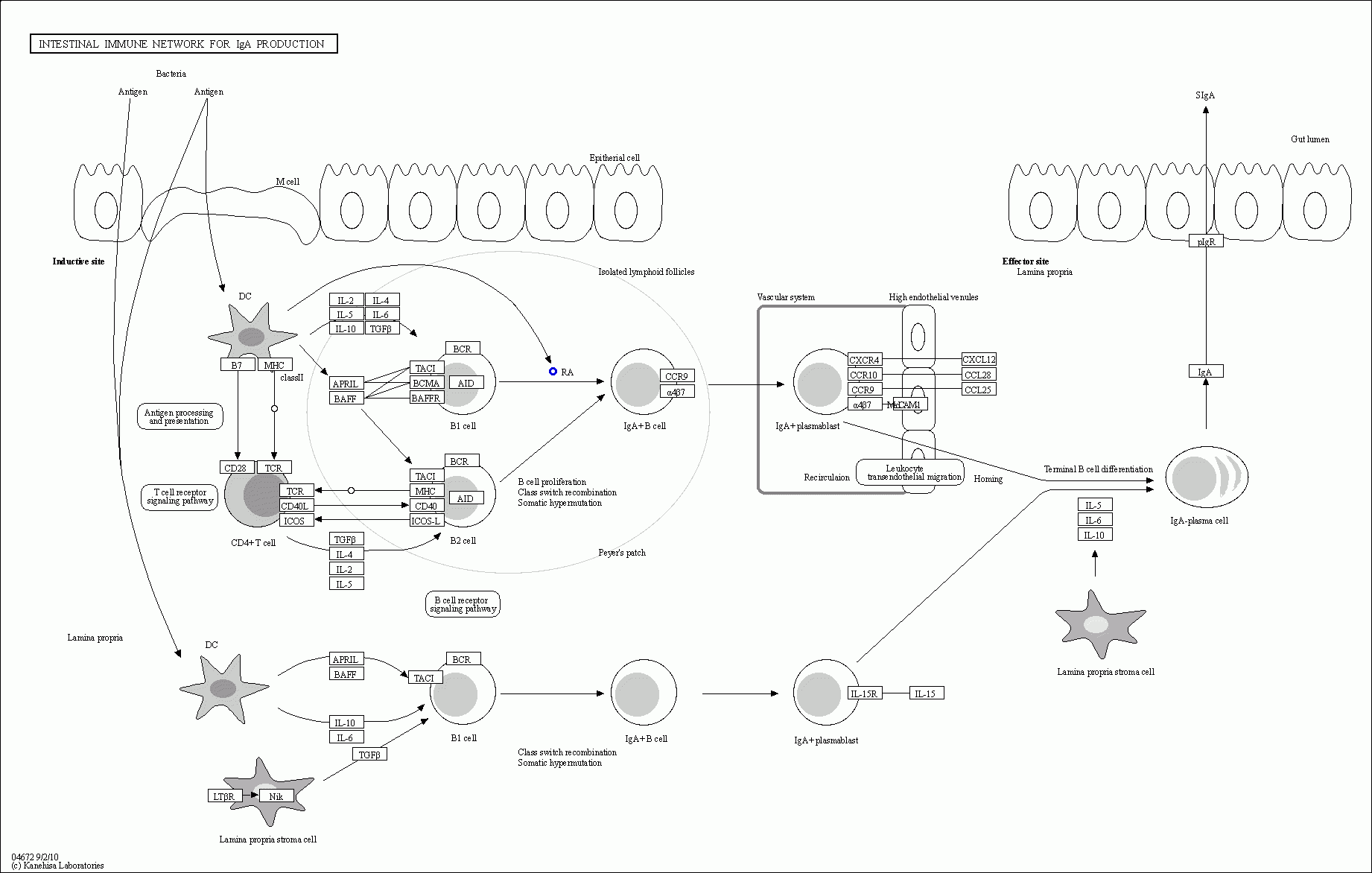

Supplement: Supplementary file 12 — Supplementary Material 12. KEGG Metabolic Enrichment Pathway. [file 13578_2024_1253_MOESM12_ESM.png]
